# Chikungunya Beyond the Tropics: Where and When Do We Expect Disease Transmission in Europe?

**DOI:** 10.3390/v13061024

**Published:** 2021-05-29

**Authors:** Nils Benjamin Tjaden, Yanchao Cheng, Carl Beierkuhnlein, Stephanie Margarete Thomas

**Affiliations:** 1 Department of Biogeography, University of Bayreuth, D-95447 Bayreuth, Germany; nils.tjaden@uni-bayreuth.de (N.B.T.); Yanchao1.Cheng@uni-bayreuth.de (Y.C.); carl.beierkuhnlein@uni-bayreuth.de (C.B.); 2 Bayreuth Center of Ecology and Environmental Research BayCEER, University of Bayreuth, D-95447 Bayreuth, Germany

**Keywords:** chikungunya, mosquito-borne disease, dengue, ecological niche model, epidemiological model, *Aedes albopictus*

## Abstract

Chikungunya virus disease (chikungunya) is a mosquito-borne infectious disease reported in at least 50 countries, mostly in the tropics. It has spread around the globe within the last two decades, with local outbreaks in Europe. The vector mosquito *Aedes albopictus* (Diptera, Culicidae) has already widely established itself in southern Europe and is spreading towards central parts of the continent. Public health authorities and policymakers need to be informed about where and when a chikungunya transmission is likely to take place. Here, we adapted a previously published global ecological niche model (ENM) by including only non-tropical chikungunya occurrence records and selecting bioclimatic variables that can reflect the temperate and sub-tropical conditions in Europe with greater accuracy. Additionally, we applied an epidemiological model to capture the temporal outbreak risk of chikungunya in six selected European cities. Overall, the non-tropical ENM captures all the previous outbreaks in Europe, whereas the global ENM had underestimated the risk. Highly suitable areas are more widespread than previously assumed. They are found in coastal areas of the Mediterranean Sea, in the western part of the Iberian Peninsula, and in Atlantic coastal areas of France. Under a worst-case scenario, even large areas of western Germany and the Benelux states are considered potential areas of transmission. For the six selected European cities, June–September (the 22th–38th week) is the most vulnerable time period, with the maximum continuous duration of a possible transmission period lasting up to 93 days (Ravenna, Italy).

## 1. Introduction

Chikungunya virus disease (chikungunya) is a mosquito-borne infectious disease, reported in at least 50 countries all over the world [1]. The disease manifests itself in a sudden rise in fever, myalgia, arthralgia, headaches, rashes, and chronic arthritis [2]. Chikungunya is caused by the chikungunya virus (CHIKV), a single-stranded positive RNA-enveloped virus (*Alphavirus*, Togaviridae), which was first isolated in a Tanzanian outbreak in 1952 [3]. In the urban transmission cycle, which occurs in Africa, America, Asia, and Europe, CHIKV is mainly transmitted from human to human by the mosquitoes *Aedes aegypti* (yellow fever mosquito) and *Ae. albopictus* (Asian tiger mosquito) [4]. Vertical transmission during childbirth (perinatal) also occurs regularly, often leading to severe central nervous system disease that can be fatal [5,6]. Vertical transmission has also been reported among mosquitoes [7]. In Africa, the transmission is maintained in a sylvatic cycle involving *Aedes* mosquitoes as vectors and primates (and probably also rodents) as hosts [4].

After the first isolation of the chikungunya virus during the 1952–53 epidemic in today’s Tanzania [3], chikungunya was mostly restricted to local outbreaks in Africa and southeastern Asia, with *Ae. aegypti* acting as the main vector [8]. Within the past two decades, however, chikungunya has spread around the globe, increasing its geographical range particularly in sub-Saharan Africa, southern and south-eastern Asia, the Western Pacific regions, and most recently in Latin America, including the Caribbean [8,9,10]. In addition to spatially limited local outbreaks, large and long-lasting epidemics are emerging as well [11,12]. In the 2005–06 outbreak in India, nearly 1.4 million cases were reported [13]. In 2006–07, about one-third of the population of La Reunion (i.e., approx. 250,000 people) were affected [14] by a new variant of CHIKV. This variant showed a mutation that led to more efficient dissemination in *Ae. albopictus*, enabling the species to act as the main vector in an outbreak situation for the first time [15]. After CHIKV arrived in the Caribbean in 2013, at least 2.6 million suspected chikungunya cases were reported from this region until the end of 2017 [16], and CHIKV has subsequently spread to large parts of South America [17]. Outbreaks followed, for example, in Kenya [18], Bangladesh [19], Pakistan [20], Sudan [21], and Nepal [22]. The geographic range and frequency of epidemics and outbreaks are globally increasing [23].

In Europe, the first local outbreak of chikungunya was reported in 2007 in Italy (Emilia-Romagna region), with 205 cases (either confirmed in the laboratory or defined clinically) [24]. Since then, chikungunya outbreaks have occurred repeatedly at the local scale. In France, two confirmed cases were recorded in 2010 (Var department) [25], and another 12 cases were confirmed in 2014 (Montpellier) [24,25,26,27]. The first known transmissions of the chikungunya virus in central and southern Italy (in the regions Lazio and Calabria) were documented in 2017, with 270 confirmed and 229 suspected cases overall [28,29,30,31,32]. In the same year, two transmission clusters in France (Var department) led to 15 confirmed and two suspected cases [28,33,34].

The primary vector species in mainland Europe is *Aedes albopictus*, as *Ae. aegypti* is currently only found on the island of Madeira (Portugal) and in the Black Sea region [35]. *Ae. albopictus* originated in southeast Asia. Since it was introduced to southern Europe by human-mediated long-distance travel (e.g., transport of used tires and ornamental plants) about three decades ago, it has considerably expanded its range there [36]. At present, *Ae. albopictus* has established populations in large areas in southern Europe, and its range expansion is ongoing. Medium-distance travel (e.g., trucking, private tourism) is relocating individuals towards the central parts of Europe [37]. This leads to increasingly frequent introductions into previously *Ae. Albopictus*-free regions, followed by instant population establishments [38]. Climate change also affects the distributional range of mosquitoes. For instance, with increasing minimum temperatures or extended warm seasons, some currently unsuitable areas may turn into suitable climatic conditions for mosquitoes [39,40,41].

In the face of the globally increasing transmission of CHIKV and the expanding spatial range of mosquito vectors, prevention measures need to be undertaken before an outbreak occurs. To prevent an outbreak, it is important to know where and when a chikungunya outbreak could potentially occur. One common approach for the creation of spatial risk maps is the application of ecological niche models (ENMs). These correlation-based models estimate the geographic distribution of a disease based on known locations of disease occurrence and the prevailing environmental conditions at those locations [42,43]. The ENM approach has been successfully applied for several mosquito-borne diseases, such as dengue [44], Zika [45], and Japanese encephalitis [46]. For chikungunya, two global ENMs have previously been published [47,48]. These two global models generally predict the observed large-scale spatial distribution patterns of chikungunya well. However, neither of them adequately reflects the situation at the borders of the current spatial distribution. In Europe, they both dramatically underestimate the potential for chikungunya outbreaks. The same is true for dengue, where the global model [44] fails to predict real-life outbreaks in Europe [49]. These three global models for chikungunya and dengue were all built upon occurrence locations that are predominantly located in the tropics. Consequently, it is not surprising that they do not perform well in temperate or Mediterranean climates, because seasonal dynamics differ vastly, with tropical climates being dominated by precipitation patterns rather than temperature seasonality. Factors such as extreme frost events that can affect the survival of mosquito eggs [39] do not play a major (if any) role in the tropics. To obtain meaningful chikungunya risk maps for non-tropical regions like Europe, models need to be calibrated with data from regions that have a more similar climate compared with the target area.

To assess where chikungunya can be transmitted in Europe with greater accuracy, we present a new, “non-tropical” ENM, built upon the global distribution of CHIKV transmission outside of the tropics. We directly compare this model with a previously published global ENM for chikungunya [48]. We further employ an epidemiological (i.e., processes-based) model that was recently proposed for Canada [50] to a series of European cities to evaluate the correlative model and add the temporal dimension for CHIKV risk assessment in non-tropical climates.

The new non-tropical model predicts real-life CHIKV transmission in Europe with greater accuracy than the previous global ENMs that were based on predominantly tropical occurrence locations. It also shows that areas at risk of CHIKV transmissions in Europe are much more widespread than previously anticipated.

## 2. Materials and Methods

### 2.1. Ecological Niche Models

Ecological niche models (ENMs) are widely used in ecology, biogeography, and conservation biology to assess the potential spatial distribution of species or other taxa [51,52,53,54]. In epidemiology, ENMs are often used to model the distribution of disease vectors or vector-borne disease transmission [42,43]. Building upon a previously published global chikungunya model [48], we have created an ENM for CHIKV transmission outside the tropical zones. For this task, we applied the most commonly used ENM approach [43], Maxent (version 3.3.3.k [55]). Maxent is a maximum entropy-based machine-learning approach, which relates species occurrence records to spatial representations of relevant environmental predictor variables. Based on this, a map of relative “environmental suitability” (ranging from 0 to 1) was created, in which higher values indicate more favorable environmental conditions, and thus a higher probability of occurrence.

#### 2.1.1. Occurrence Records

The global database of geo-referenced locations of CHIKV transmission described by Tjaden et al. [48] was updated to include a total of 845 records. Each of these records represents a local disease event with at least one case of autochthonous transmission. As infectious diseases are typically reported for entire administrational units, the coordinates of the geographic centroid of these units were used as records unless a more precise description of the place of transmission was provided. These records were then refined through a series of filters. Firstly, records located in any of the tropical climate zones (Köppen–Geiger classification Af, As, Am, or Aw) were discarded (see Figure S1). For this, the Köppen–Geiger climate classification was obtained from [56] (representative of the 36 years between 1980–2016). Secondly, 15 more records from the Andean region were removed manually. These were centroids of large (provincial-level) administrational units located in a highly heterogeneous environment, and were thus likely to negatively affect the model’s performance [57]. Finally, the spThin package version 0.2.0 [58] for R 3.6.1 [59] was used to spatially rarefy the occurrence records. A basic assumption of ENMs is that the study area is sampled evenly. The unrarefied data set violates this assumption due to variations in international reporting patterns. For the thinning parameter of the thin function, values between 5 and 20 were considered and the resulting spatial clustering was visually assessed. Ultimately, the package’s default value of 10 was used, as this setting resolved obvious clusters without removing valuable occurrence records elsewhere. For model evaluation, the final 160 occurrence records (Figure S1) were randomly split into a 10-fold cross-validation.

#### 2.1.2. Environmental Data

The standard set of 19 bioclimatic variables was used in this study to ensure comparability with the 2017 global model [48]. However, the data source of those variables was updated to use Worldclim version 2.1 [60] at a spatial resolution of 2.5′ instead of the now-outdated version 1.4. Like the occurrence records, the environmental raster layers were clipped by climate zone, discarding all data located in the tropical regions (Köppen–Geiger classification Af, As, Am, or Aw [56], Figure S1).

#### 2.1.3. Calibration Area

In addition to occurrence locations, Maxent also allocates background (or: “pseudo-absence”) locations within a defined geographical range of the environmental data. In the default setting, the geographical range is the whole available area. The geographical range, the area from which Maxent is allowed to draw background locations, can affect model performance and results [61]. Consequently, it needs to be defined carefully. Following VanDerWal (2009) [61], we opted for a buffer-based approach that was successfully employed in other studies with similarly large geographic extents [48,62]. A series of buffers with increasing radii (from 1 to 5000 km) drawn around the occurrence records was used, and the environmental data were cropped accordingly. Based on these differently sized calibration areas, we ran a series of test Maxent models, and evaluated model performance with true skill statistics (TSS) [63]. Model performance increased with the buffer size, but the gain in TSS considerably slowed down when the buffer reached a radius of 1500 km. Consequently, a 1500-km buffer was used to delineate the study region for the subsequent model runs (see Figure S1).

#### 2.1.4. Model Selection

Model selection, i.e., selecting the environmental predictor variables to be used as well as the model settings (choice of regularization multiplier and feature types for Maxent [64]), was performed following an incremental approach. First, a reference model using the whole suite of bioclimatic variables available in the Worldclim 2.1 dataset was built. The optimum calibration area was determined as described above. The model settings were optimized based on Akaike’s information criterion (AIC) scores using ENMEval version 0.3.0 [65] for R 3.6.1. Afterwards, a series of candidate models with different sets of variables was built in the same way, and their performance was evaluated based on AIC, TSS [63], area under the receiver operating characteristic curve (AUC), and their ability to predict the European outbreak locations as “environmentally suitable”.

Candidate variable sets were constructed by dropping variables from the full set based on the following criteria. (1) It has been shown that the influence of precipitation on mosquito populations is anything but straightforward. For instance, the container-breeder *Ae. albopictus* frequently uses artificial habitats, such as flowerpots and vases, that are independent of precipitation [66]. In addition, although precipitation is generally considered beneficial, heavy rainfall events can have adverse effects on larval mosquito populations [67,68]. Hence, we considered a variable set without any precipitation-related variables. (2) Variables that refer to the wettest or driest month or quarter of the year do not translate well across different regions. For instance, preliminary analyses showed that the variable “temperature of the wettest quarter” refers to temperatures in summer and winter at different locations in Europe. A variable that bears different meanings in different places needs to be considered as potentially problematic. Hence, we investigated whether dropping these variables would improve the model. (3) Variables such as the “mean diurnal temperature range” or “precipitation seasonality” do not strictly represent temperature or precipitation, but rather represent values derived from them. We considered dropping these variables, as they add another potentially detrimental layer of complexity to the model (compare, e.g., [69,70]). (4) Additionally, we used the built-in jackknife procedure in Maxent to remove variables that did not contribute useful information to any of the models [71]. For reference, the variable set from the 2017 global model was reproduced as well.

The final model was based on the combination of criteria (3) and (4), using the annual mean temperature, minimum temperature of the coldest month, annual temperature range, mean temperatures of the warmest and coldest quarters, annual precipitation sum, as well as the precipitation of the warmest quarter (see Table S1 for details). It uses linear, quadratic, and hinge features, with a regularization multiplier of 4. This final model configuration was evaluated using partial ROC testing [72] as implemented in the ENMGadgets package version 0.1.0.1 [73] for R 3.6.1. This was based on a 10-fold cross-validation run of Maxent, using 1000 bootstrapping iterations with an expected error rate of 5%. All AUC ratios were larger than 1. Together with an average test AUC of 0.89 and a TSS value of 0.814, this suggests good model performance. The transferability of the model into new regions outside the calibration area was evaluated using the multivariate environmental similarity surface functionality available in Maxent [74]. No areas of strict extrapolation occurred across Europe (Figure S2), suggesting good transferability in space.

#### 2.1.5. Thresholds

The relative environmental suitability predicted by Maxent is a series of continuous values. These raw values alone are not sufficient to decide whether a place should be considered unsuitable for disease transmission. To achieve a map of suitable vs. unsuitable areas, a threshold that can classify the raw values into binary results is needed. For this purpose, several methods have been proposed [75,76]. As there is no single best-practice method (and to facilitate comparisons across different studies), we applied a series of commonly used thresholds.

The “minimum training presence” threshold was used to show a worst-case scenario. This simple method is also called the “lowest presence threshold” and focuses on the values of environmental suitability predicted for the locations where CHIKV transmission occurred [77]. Among these locations, the one with the lowest predicted suitability is identified, and this lowest suitability value is used as the threshold. A more conservative approach is first to discard the 5 or 10 percent of occurrence records that have the lowest predicted environmental suitability and use the minimum of the remainder as a threshold [77]. These are referred to as the 5- or 10-percentile thresholds, respectively. Finally, the “equal sensitivity and specificity” threshold aims for a balance in the trade-off between sensitivity and specificity [78]. It presents the most conservative estimate of CHIKV transmission for our models, highlighting the areas of highest risk.

### 2.2. Epidemiological Model

To assess the temporal dynamics of chikungunya transmission, as well as the credibility of the non-tropical ENM, we applied a process-based disease transmission model. We chose the chikungunya model developed by Ng et al. [50], as it was developed for the non-tropical climate of Canada and does not require external calibration through local field data. In summary, the stochastic model of [50] calculates the basic reproduction number R_0_ as:

R_0_ = φ ∙ α^2^ ∙ β_HM_ ∙ β_MH_ ∙ L ∙ V ∙ γ. (1)

From a theoretical point of view, R_0_ represents the typical number of secondary infections that arise from a single infected individual throughout its infectious period in a completely susceptible population. This is a threshold quantity: when R_0_ > 1, the disease can spread, otherwise it will die out. The daily biting rate (α), human-to-mosquito and mosquito-to-human transmission probabilities (βHM, βMH), and the duration of the human infectious period (V) are static parameters that are estimated based on a probability distribution for each iteration of a model run. Adult mosquito life span (L), the fraction of mosquitoes surviving the extrinsic incubation period (γ), and the mosquito density per human (φ) additionally depend on temperature (see Table S2 for full parameterization). The model was implemented in R 3.6.1 [59] and run at 50,000 iterations to account for uncertainties in the parameter estimations.

We selected a series of reference locations across the study area for which the model was run. Aiming for a small but representative sample, we included areas of previous CHIKV transmission, as well as areas with different levels of climatic suitability as predicted by the non-tropical ENM. Daily mean temperature data for these locations (see Figure S3 for an overview) were obtained from nearby weather stations, using the blended European Climate Assessment (ECA) data set [79]. For each reference location, three time series of daily mean temperature data were extracted for three model runs: (1) the year 2018, as an extreme example with an unusually hot summer in large parts of Europe; (2) 2017, a year with outbreaks in Italy and France; and (3) the long-term average of the years 1970–2000, which corresponds to the reference period used in the Worldclim data set upon which the non-tropical ENM was built. Small gaps in the data (≤5 days) were filled through cubic spline interpolation [80]. Larger gaps were filled by extracting additional data from E-OBS, a spatio-temporally interpolated version of the ECA data set [81] (Figure S3). This way, a complete time series of daily mean temperature was available for all but one reference locations.

The estimated R_0_ values of the 50,000 iterations per model run were aggregated into a single average value per day. After the number of days with an average R_0_ ≥ 1 was determined, values were further aggregated into weekly averages for plotting.

## 3. Results

**Figure 1:**
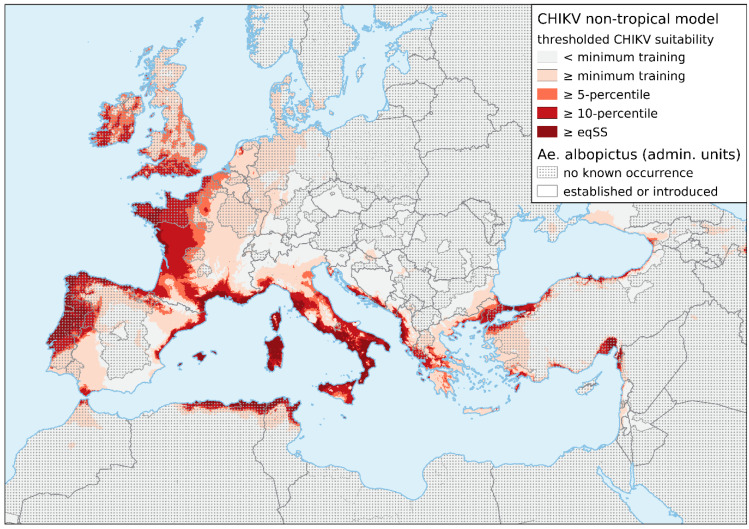
Categorized environmental suitability map of CHIKV transmission under current climatic conditions, based on an ecological niche model of 160 global chikungunya case localities outside the tropics. Explanatory bioclimatic variables were derived from Worldclim (http://worldclim.com accessed on 28 May 2021). The predicted potential transmission was classified into binary results according to a series of thresholds—minimum training: the place with the lowest suitability where CHIKV transmission occurred; 5/10-percentile: 5 or 10 percent of occurrence records that have the lowest predicted environmental suitability are discarded, and the lowest remaining values mark the threshold; eqSS: “equal sensitivity and specificity” threshold. The current distribution of the invasive mosquito *Ae. albopictus* in the EU/EEA at regional administrative unit level NUTS 3 was derived from the maps provided by the European Centre for Disease Prevention and Control (ECDC) as of March 2021 [82]. Established: evidence of reproduction and overwintering has been observed in at least one municipality within the administrative unit. Introduced: the species has been detected (but without confirmed establishment).

The non-tropical ENM predicts large parts of southern and western Europe and the coastal regions of south-eastern Europe to be suitable for chikungunya transmission (Figure 1). In areas where the vector is known to be established, the highest climatic suitability for chikungunya transmission is projected along with the northern and eastern coastal areas of the Mediterranean Sea and the Atlantic-influenced areas. This primarily affects mainland Portugal, Spain, France, Italy, Croatia, Albania, and Greece; several touristic islands, including the Baleares, Corse, Sardinia, and Crete; as well as the southern coastal areas of the Black Sea. The coasts of northern Italy, Turkey, Israel, and Lebanon show a medium risk, only passing the minimum training or 5-percentile thresholds.

Large areas with the highest climatic suitability for chikungunya transmission can also be found in areas where *Ae. albopictus* is currently not known to be established (large parts of Portugal, Ireland, and southern England) or not monitored (northern parts of Spain and large parts of the African coastal areas of the Mediterranean Sea).

It is noteworthy that even areas that are considered to be at low risk for chikungunya have seen local outbreaks in the past. For example, the Emilia-Romagna region, in Italy, in which the first CHIKV transmission in Europe took place, only passes the minimum training gain threshold. This means that transmission may potentially occur in other regions with low climatic suitability, such as central Spain, northern France, the Benelux states, Germany, Croatia, Montenegro, Albania, and Greece.

**Figure 2:**
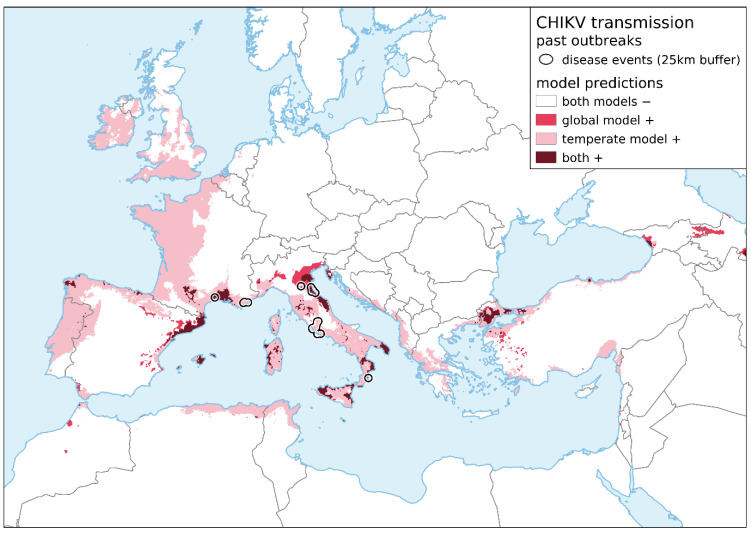
Comparison of environmental suitability as determined by the global model [48] and the non-tropical model under current climatic conditions. Results from both models were transformed into binary maps according to the 5-percentile threshold and compiled through a map overlay. Autochthonous transmissions of chikungunya are displayed as black polygons (25-km buffer zone around occurrence locations).

When compared with the global model [48], the non-tropical model predicts all European outbreaks with greater accuracy (Figure 2; see also Figure S3 for place names). The global model fails to project the later outbreaks in 2014 (Var, France) and 2017 (Lazio and Calabria regions, Italy), whereas the non-tropical model covers both outbreaks. Both global and non-tropical models correctly predict Montpellier (France) and Ravenna (Italy) as areas at risk for CHIKV transmission. For Bologna (Italy), however, only the global model projects it as at risk.

Overall, the global model only predicts a few small and scattered patches to be suitable for CHIKV transmission in Europe. The largest area predicted to be suitable by this model is located in northeastern Italy. In several small patches in southern Italy, Montpellier (southern France), Mallorca, southern Spain, and the Marmara Region (Turkey), the global model overlaps with the non-tropical model, which shows considerably more continuous predictions. There are small regions in Georgia and Azerbaijan that are classified as suitable in the global model that are not considered suitable in the non-tropical model.

**Figure 3:**
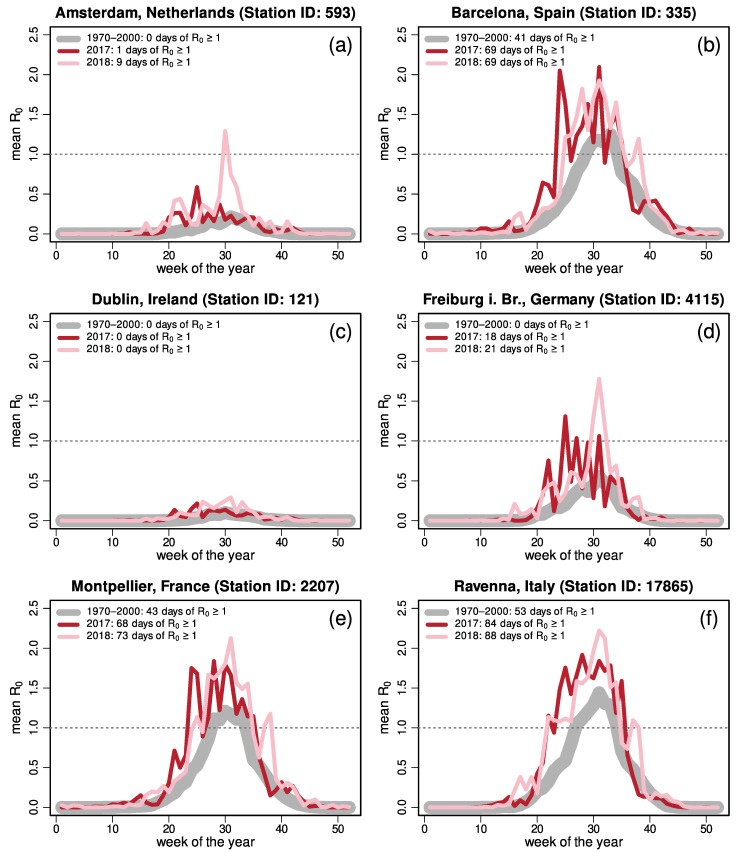
Potential chikungunya outbreak risk in six cities in Europe, based on the epidemiological model (EM) as assessed through the temporal development (weekly averages) of the basic reproduction number, R_0_. The thick gray line represents calculations based on long-term average values of daily mean temperature during the Worldclim reference period (1970–2000). The thin red and pink lines show single-year values for 2017 and 2018, respectively. The individual cities reflect different classes of spatial risk projections in the non-tropical ecological niche model (ENM) and temporal risk assessment based on the EM: (**a**) Amsterdam is located in an area at risk according to both of the ENMs, but the EM shows a few days of R_0_ ≥ 1 in 2018; (**b**) Barcelona is at risk according to both models, with a high transmission potential predicted by the EM; (**c**) Dublin is at risk according to the non-tropical model, but the EM suggests no potential for transmission; (**d**) Freiburg is not in an area at risk in either ENM, but shows a temporal risk in both years; (**e**) Montpellier and (**f**) Ravenna are at risk according to all applied models.

The epidemiological model provides a different perspective on the CHIKV transmission potential in Europe (Figure 3 and Figure S5; see also Figure S1 for locations). For short-term risk fluctuations, Freiburg (Germany, (d)) displays one high-risk peak, with a total of 21 days at risk in 2018. Amsterdam (the Netherlands, (a)) shows a low risk for this year, with 9 days with R_0_ ≥ 1. For 2017, Freiburg (but not Amsterdam) shows a short period of outbreak risk, of 18 days. The EM suggests no potential for transmission in Dublin for both years. For the three southern European cities, i.e., Barcelona (Spain, (b)), Montpellier (France, (e)), and Ravenna (Italy, (f)), it appears that all have a risk of chikungunya transmission that persists up to 10 to 12 weeks during summer. It should be noted that in both Montpellier and Ravenna the autochthonous transmission of chikungunya has occurred in the past [24].

## 4. Discussion

In this study, a non-tropical ENM was applied to assess the spatial outbreak risk of chikungunya in Europe. The results were compared with those of a global model [48] and validated against an epidemiological model that was applied for six representative cities within the study area. In addition, this epidemiological model captures the temporal outbreak risk.

The non-tropical model projects all past outbreaks in Europe. It thus reflects real-life transmission much more accurately than either of the previously published ENMs for chikungunya, which predicted very small [48] or no [47] climatically suitable areas in Europe. Compared with those previous models, the non-tropical model predicts considerably larger at-risk areas of chikungunya transmission in western and central Europe (eastern France, southern England, and Ireland), regions in southern Europe (western Spain and northern Italy), and south-eastern Europe (Greece and western Turkey). Using the sensitive “minimum training presence” threshold as a worst-case scenario, even large areas of western Germany and the Benelux states may be considered potential areas of transmission. The temporal risk assessments for 2003 and 2019, given by the epidemiological model for cities in these areas, indeed indicates 23–60 days of R_0_ ≥ 1 for Freiburg in south-western Germany, but only a very short time-window of transmission for Amsterdam (7–9 days of R_0_ ≥ 1). This confirms that relying on a single type of model is not sufficient for the risk-assessment of vector-borne diseases (compare, e.g., [43,62]). At this point, it is again important to mention that the ENM includes both temperature- and precipitation-based variables, whereas the EM is purely based on daily mean temperatures. This dichotomy between the ENM and the epidemiological model is very pronounced in Ireland. There, the non-tropical ENM predicts high climatic suitability for CHIKV transmission, whereas the epidemiological model estimates 0 days of R_0_ ≥ 1 for all the time series under consideration. This can be explained, at least partially, through Ireland’s oceanic climate. The absence of frost events in the mild winters facilitates mosquito survival [39], whereas summer temperatures are not high enough to facilitate disease transmission. Further factors, such as salinity or wind speed, that could affect vector distribution [83,84] were not included in the ENM either. The epidemiological model, on the other hand, currently assumes a fixed probability distribution for the vector-to-host ratio, not accounting for spatial differences in human population density and mosquito abundance.

Especially for temperate and subtropical regions, further epidemiological models have been developed for outbreaks in Italy [85,86,87,88], Japan [89], and the US [90]. Coupling an epidemiological model with a temperature-dependent population model of *Ae. albopictus*, Poletti et al. [85] found the same at-risk time period for the Italian provinces of Ravenna, Cesena-Forli, Rimini, and Bologna. However, since information on the actual number of breeding sites was lacking, precise estimates of the density of mosquitoes over time were not possible. For the outbreak in 2017 in Italy, Manica et al. [86] predicted the risk of chikungunya transmission in the Lazio region up until mid-November as a consequence of favorable climatic conditions. The identification of virus dispersion from outbreak foci was supported by indicators based on voluminous and velocious data in Italy and France [87], but the specificity of risk maps can be further improved by including factors such as land use and vector flight activity and biting behavior. A global model for chikungunya, dengue, and Zika transmission was established by Ryan et al. [91]. This spatio-temporal, temperature-dependent, empirically parameterized model of disease transmission by *Ae. albopictus* shows similar spatial patterns of areas at risk compared to the 5-percentile threshold of the non-tropical ENM, but misses large areas in northern parts of the Iberian Peninsula and western parts of France. In contrast, large areas of southeastern Europe are classified as seasonal (1–3 months) risk areas for chikungunya by Ryan et al. [91], which are not classified as risk areas in the ENM under any threshold.

For the ENM, one of the basic assumptions is that the target species already occupies the entirety of its environmental niche [92]. For a mosquito-borne disease, the niche consists of the joint environmental niches of its vectors, in combination with thermal effects on epidemiological parameters (such as vectorial capacity and the duration of the extrinsic incubation period; cf. [42,43]). As mentioned above, in many parts of the world, the invasive vector species *Ae. albopictus* has not yet reached its distributional boundaries. It cannot be excluded that *Ae. albopictus* either has not yet reached full niche occupancy or is currently in the process of shifting its niche [93]. In addition, global underreporting due to a lack of accurate reporting and diagnostic facilities in developing countries is a known complication when working with chikungunya case data [94,95,96]. This means that the actual niche size of chikungunya could be larger than that estimated by the ENM, resulting in an underestimation of risk. Furthermore, it needs to be kept in mind that climate change affects not only the potential at-risk region, but also the at-risk time period and the duration of the transmission season.

Climate change will further amplify the chikungunya transmission risk in Europe [91,97]. The vector *Ae. albopictus* is projected to spread further north of its current distribution [98]. Furthermore, under high carbon emission scenarios, *Ae. aegypti* is projected to invade large parts of southern Europe up to the end of this century [41,99].

The models presented here estimate the risk of chikungunya transmission based on several environmental and biological factors. Recently, it was also found that the demographic history of the invasive vector *Ae. albopictus* and co-evolution with different CHIKV strains play a specific role in the possible occurrence of autochthonously acquired chikungunya virus disease in Europe [100]. To date, all autochthonous transmissions in Europe have been caused by strains that belonged exclusively to the East/Central/South African ECSA lineage [101].

Further research is needed to elucidate the impact of biodiversity patterns (e.g., functional, species, and genetic diversity) on mosquito-borne disease transmission. Little is known, for example, about mosquito genetics, which seems to play a crucial role in CHIKV transmission [100]. Vector competence for CHIKV is suggested to be more dependent on mosquito genetics than for the dengue virus [100]. Conversely, the effects of temperature on transmission are stronger for dengue than for chikungunya [102]. The non-tropical model for Europe can be transferred to other temperate and subtropical regions which have already experienced single outbreak events to further validate the model’s results. The integration of human population density and mosquito abundance data can further narrow down areas at risk, where mosquito–host ratios are high [48]. Although the monitoring of mosquito abundance is very time-intensive, these data will be crucial in the development of abundance models which can serve as early-warning systems for mosquito-borne diseases.

## 5. Conclusions

The non-tropical model presented here expresses the real-life transmission of CHIKV in Europe with greater accuracy than the comparable previous approaches. Based on this, the estimated spatial extent of chikungunya transmission in Europe is larger than previous models have suggested. The non-tropical ENM, specifically adapted to non-tropical areas, clearly shows the areas in Europe where continuous vector monitoring is advisable, and the surveillance and reliable diagnosis of febrile illnesses should be ensured by providing information to physicians. For areas classified as high-risk, the establishment of regionally adapted early warning systems is recommended as an important means of proactive public health management. Our study proves once more that there are no “silver bullet” approaches to modeling vector-borne diseases. In practice, as all models necessarily need to simplify certain parameters of disease transmission, a single model cannot cover every aspect equally well. Consequently, it is essential to consider a variety of models, keeping in mind their strengths and weaknesses.

## Data Availability

The data presented in this study are openly in FigShare at 10.6084/m9.figshare.14696763.v1. (Link: https://figshare.com/articles/dataset/Occurrence_records_CHIKV_1996-2020/14696763/1, accessed on 28 May 2021).

## Supplementary Materials

The following are available online at https://www.mdpi.com/article/10.3390/v13061024/s1, Figure S1: Overview of the occurrence records used for the final model, regions identified as tropical, and definition of the calibration area. Figure S2: multivariate environmental similarity surface (MESS) analysis for the ecological niche model. Figure S3: Locations of weather stations used for the epidemiological models. Figure S4: Data sources and data completeness for temperature time series. Figure S5: Potential chikungunya outbreak risk in additional cities in Europe. Table S1: Variable selection for the ecological niche model. Table S2: Parameters of the epidemiological model.
