# Supplementary material for: Chikungunya Beyond the Tropics: Where and When Do We Expect Disease Transmission in Europe?
Source: Viruses. 2021 May 29;13(6):1024. doi: 10.3390/v13061024 (PMC8226708; doi:10.3390/v13061024)
Supplement: Supplementary file 1 [file viruses-13-01024-s001.zip › Figure S3.pdf]

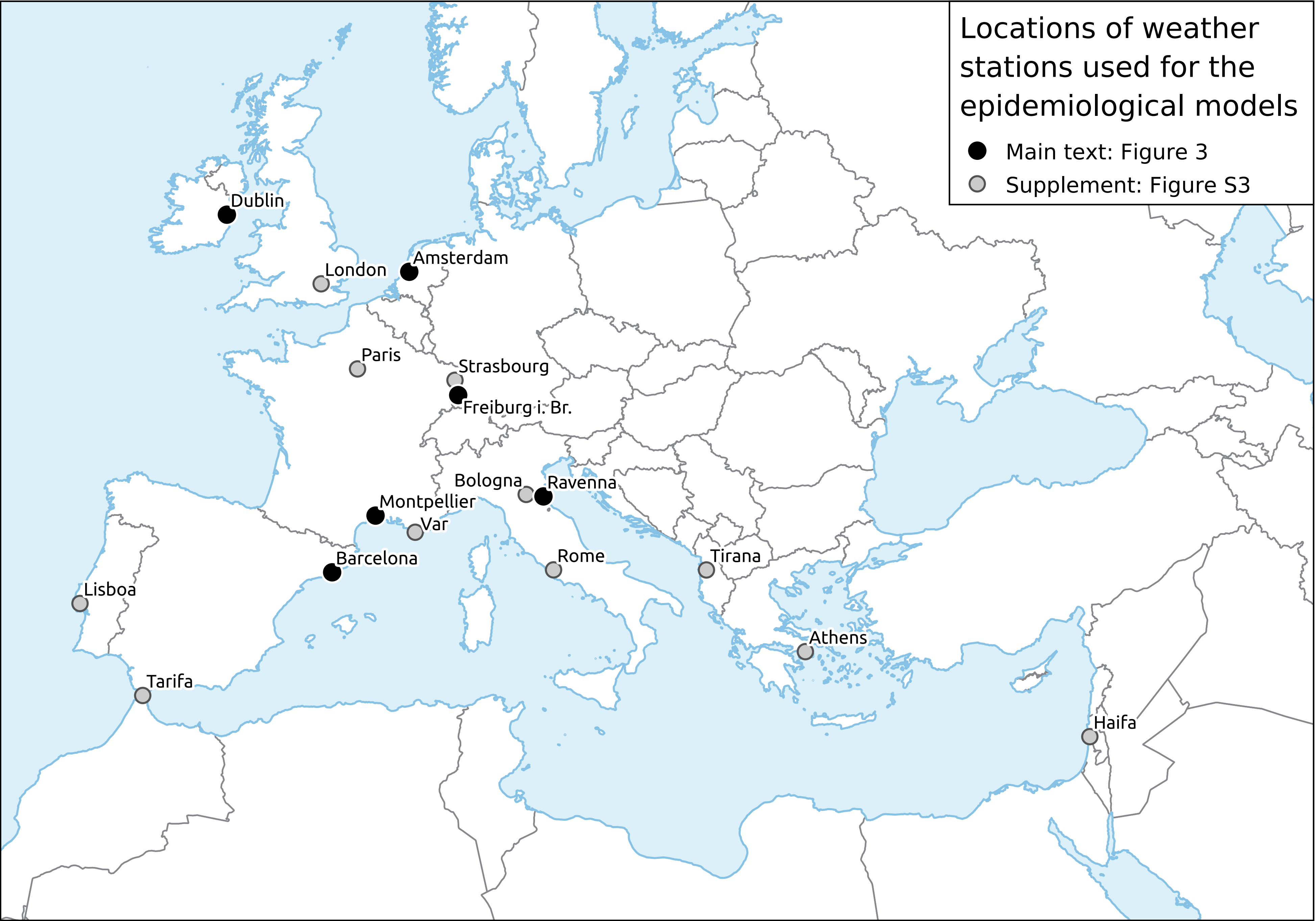

Locations of weather stations used for the epidemiological models
Main text: Figure 3
Supplement: Figure S3
Dublin
London
Amsterdam
Paris
Strasbourg
Freiburg i. Br.
Bologna
Ravenna
Montpellier
Var
Barcelona
Rome
Tirana
Lisboa
Athens
Tarifa
Haifa
