# Supplementary material for: Chikungunya Beyond the Tropics: Where and When Do We Expect Disease Transmission in Europe?
Source: Viruses. 2021 May 29;13(6):1024. doi: 10.3390/v13061024 (PMC8226708; doi:10.3390/v13061024)
Supplement: Supplementary file 1 [file viruses-13-01024-s001.zip › Figure S4.pdf]

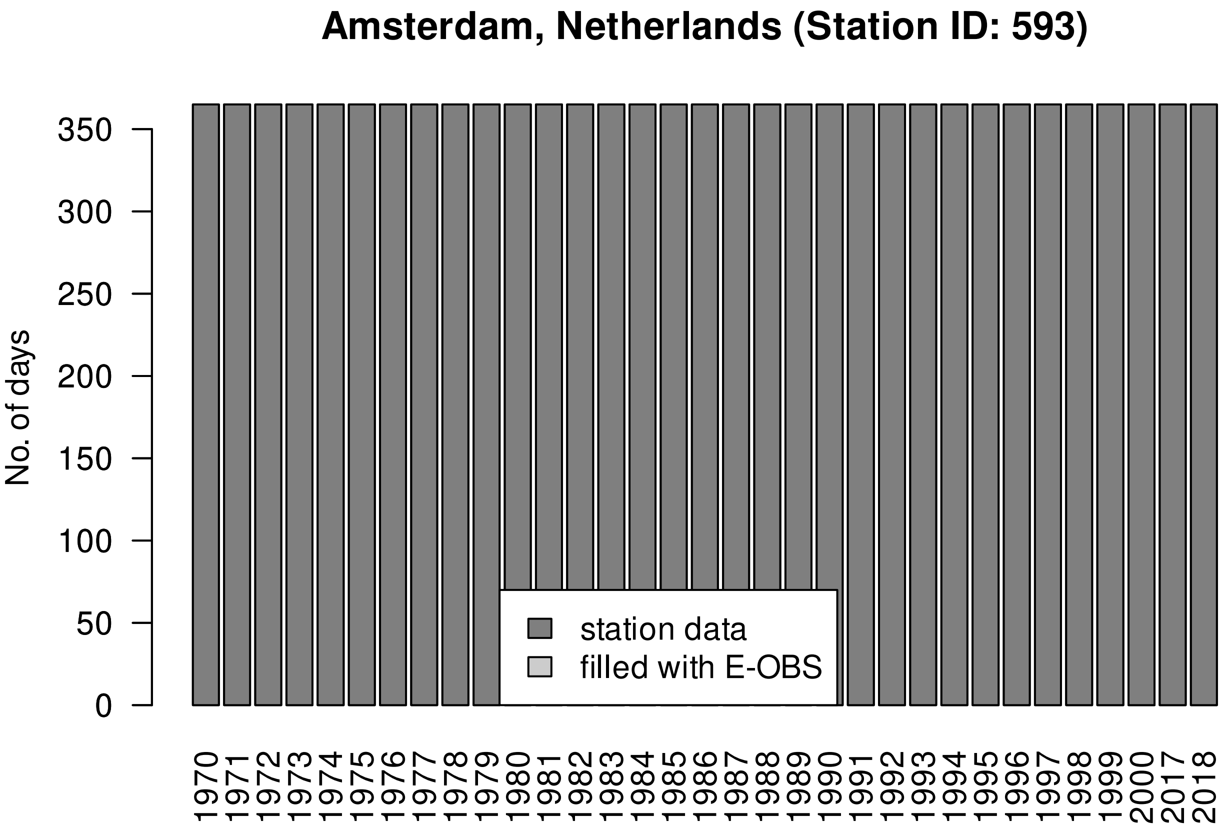
Amsterdam, Netherlands (Station ID: 593)
No. of days
0
50
100
150
200
250
300
350
station data
filled with E-OBS
1970
1971
1972
1973
1974
1975
1976
1977
1978
1979
1980
1981
1982
1983
1984
1985
1986
1987
1988
1989
1990
1991
1992
1993
1994
1995
1996
1997
1998
1999
2000
2017
2018

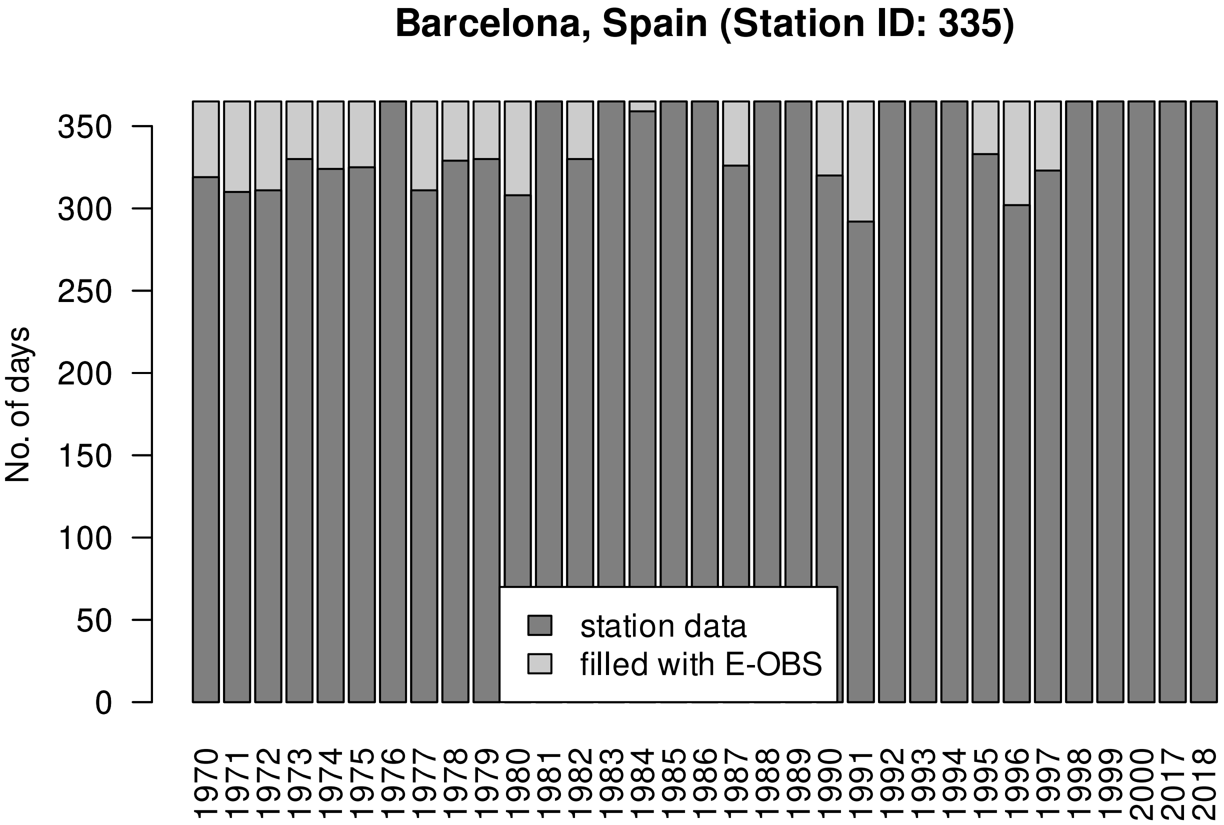
Barcelona, Spain (Station ID: 335)
No. of days
0
50
100
150
200
250
300
350
station data
filled with E-OBS
1970
1971
1972
1973
1974
1975
1976
1977
1978
1979
1980
1981
1982
1983
1984
1985
1986
1987
1988
1989
1990
1991
1992
1993
1994
1995
1996
1997
1998
1999
2000
2017
2018

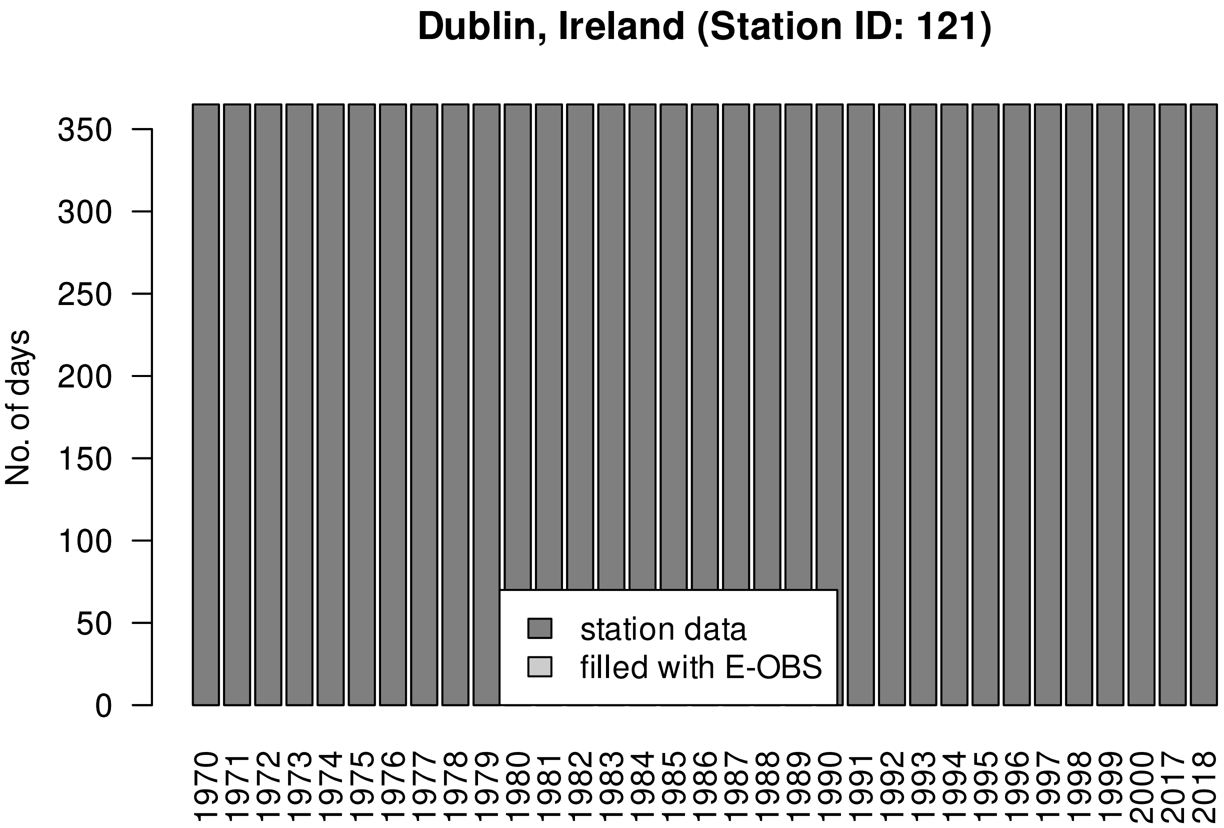

Dublin, Ireland (Station ID: 121)
No. of days
0
50
100
150
200
250
300
350
station data
filled with E-OBS
1970
1971
1972
1973
1974
1975
1976
1977
1978
1979
1980
1981
1982
1983
1984
1985
1986
1987
1988
1989
1990
1991
1992
1993
1994
1995
1996
1997
1998
1999
2000
2017
2018

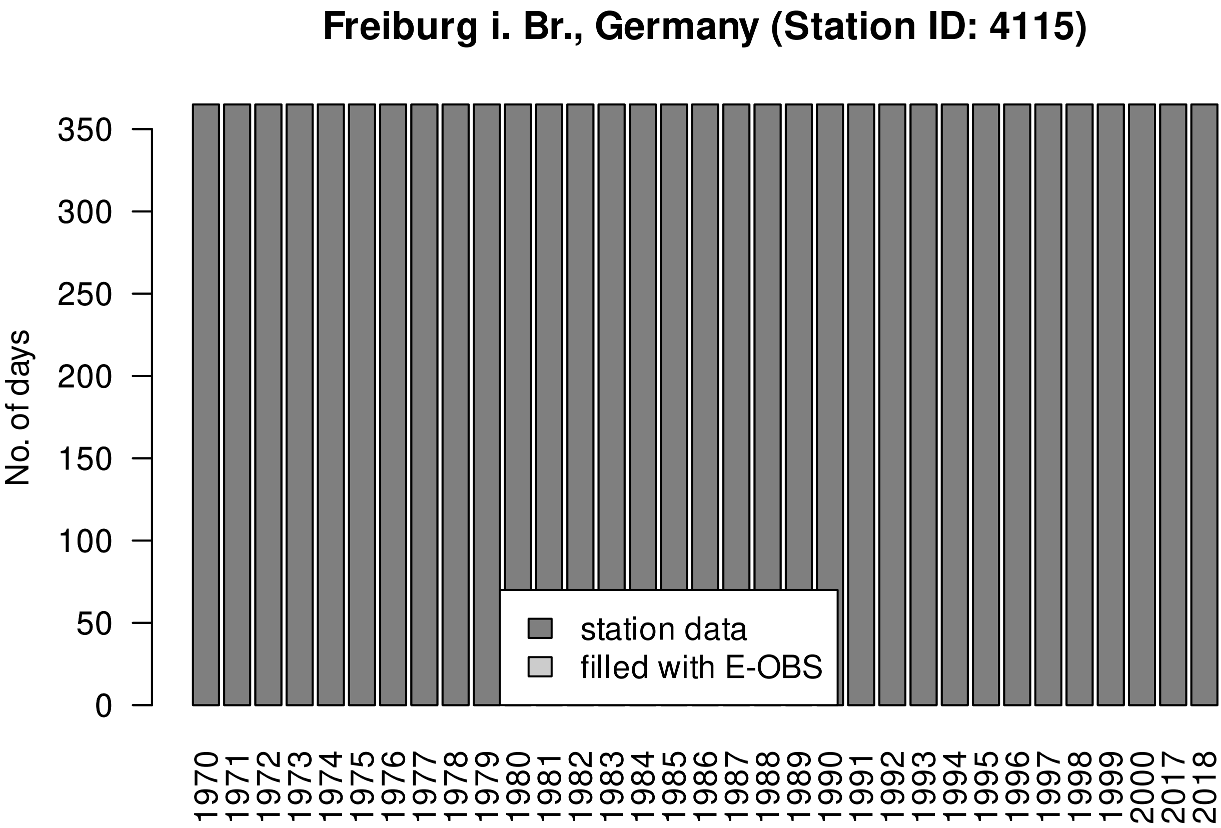
Freiburg i. Br., Germany (Station ID: 4115)
No. of days
0
50
100
150
200
250
300
350
station data
filled with E-OBS
1970
1971
1972
1973
1974
1975
1976
1977
1978
1979
1980
1981
1982
1983
1984
1985
1986
1987
1988
1989
1990
1991
1992
1993
1994
1995
1996
1997
1998
1999
2000
2017
2018

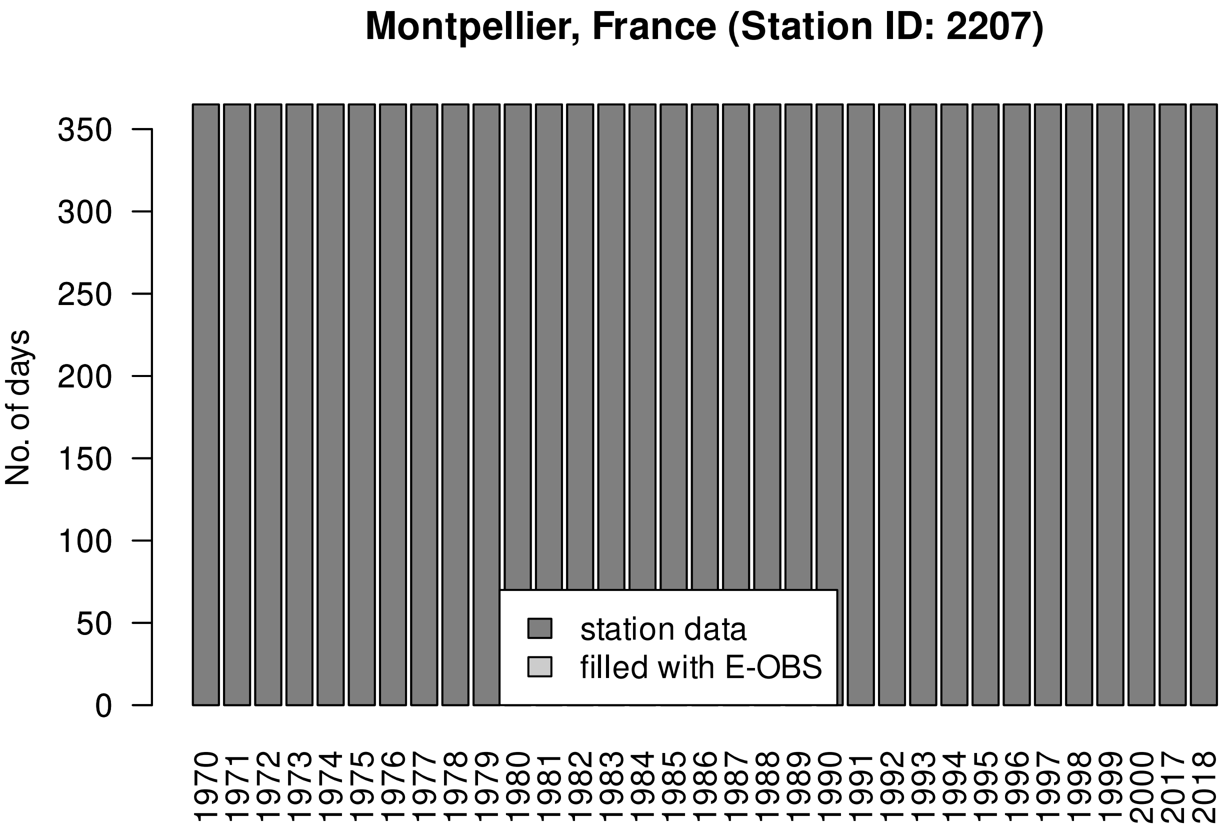

Montpellier, France (Station ID: 2207)
No. of days
0
50
100
150
200
250
300
350
station data
filled with E-OBS
1970
1971
1972
1973
1974
1975
1976
1977
1978
1979
1980
1981
1982
1983
1984
1985
1986
1987
1988
1989
1990
1991
1992
1993
1994
1995
1996
1997
1998
1999
2000
2017
2018

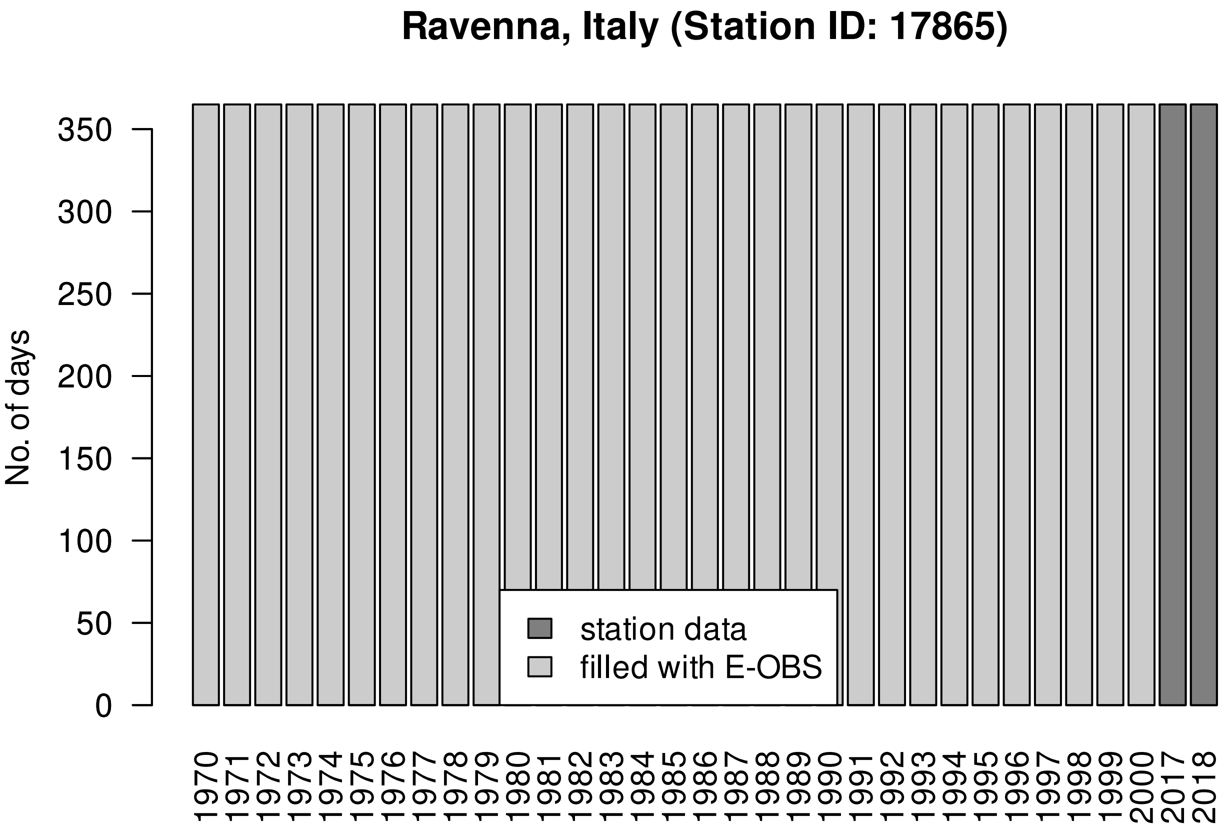

Ravenna, Italy (Station ID: 17865)
No. of days
0
50
100
150
200
250
300
350
station data
filled with E-OBS
1970
1971
1972
1973
1974
1975
1976
1977
1978
1979
1980
1981
1982
1983
1984
1985
1986
1987
1988
1989
1990
1991
1992
1993
1994
1995
1996
1997
1998
1999
2000
2017
2018

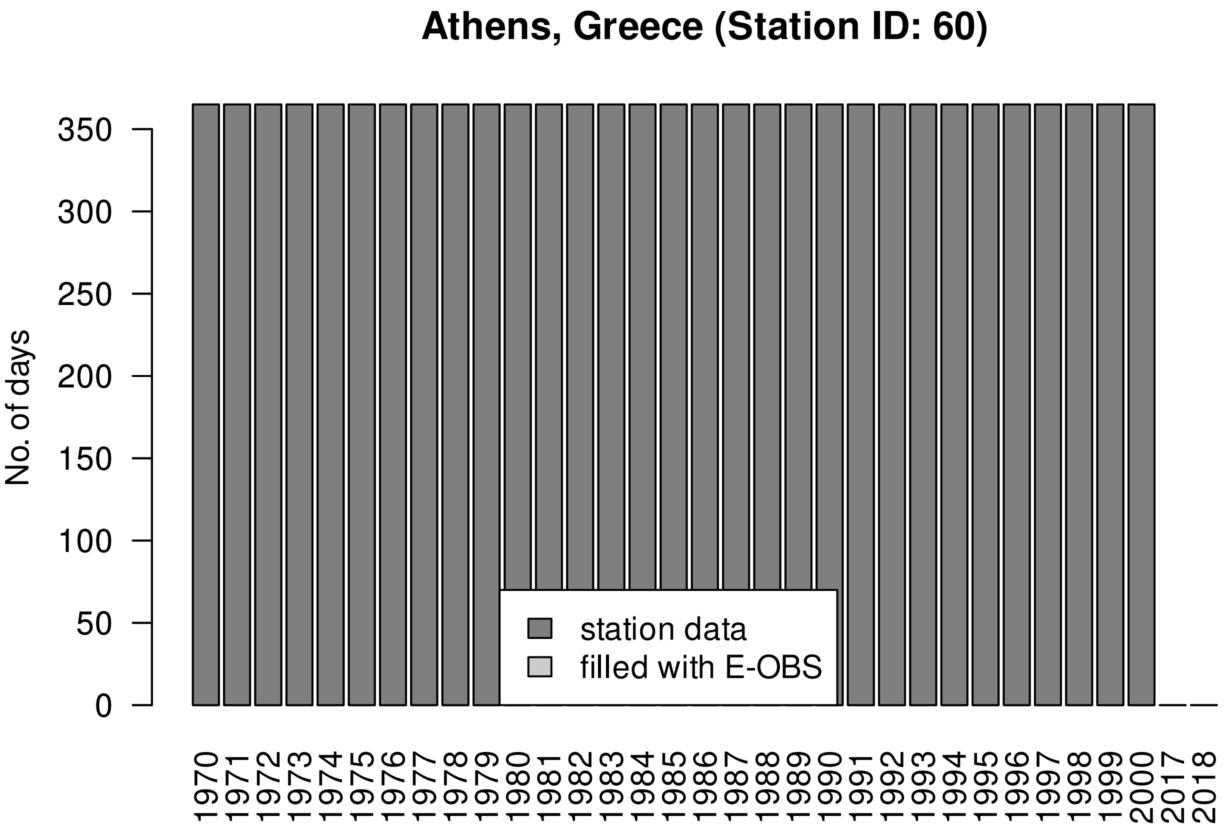
Athens, Greece (Station ID: 60)
No. of days
0
50
100
150
200
250
300
350
station data
filled with E-OBS
1970
1971
1972
1973
1974
1975
1976
1977
1978
1979
1980
1981
1982
1983
1984
1985
1986
1987
1988
1989
1990
1991
1992
1993
1994
1995
1996
1997
1998
1999
2000
2017
2018

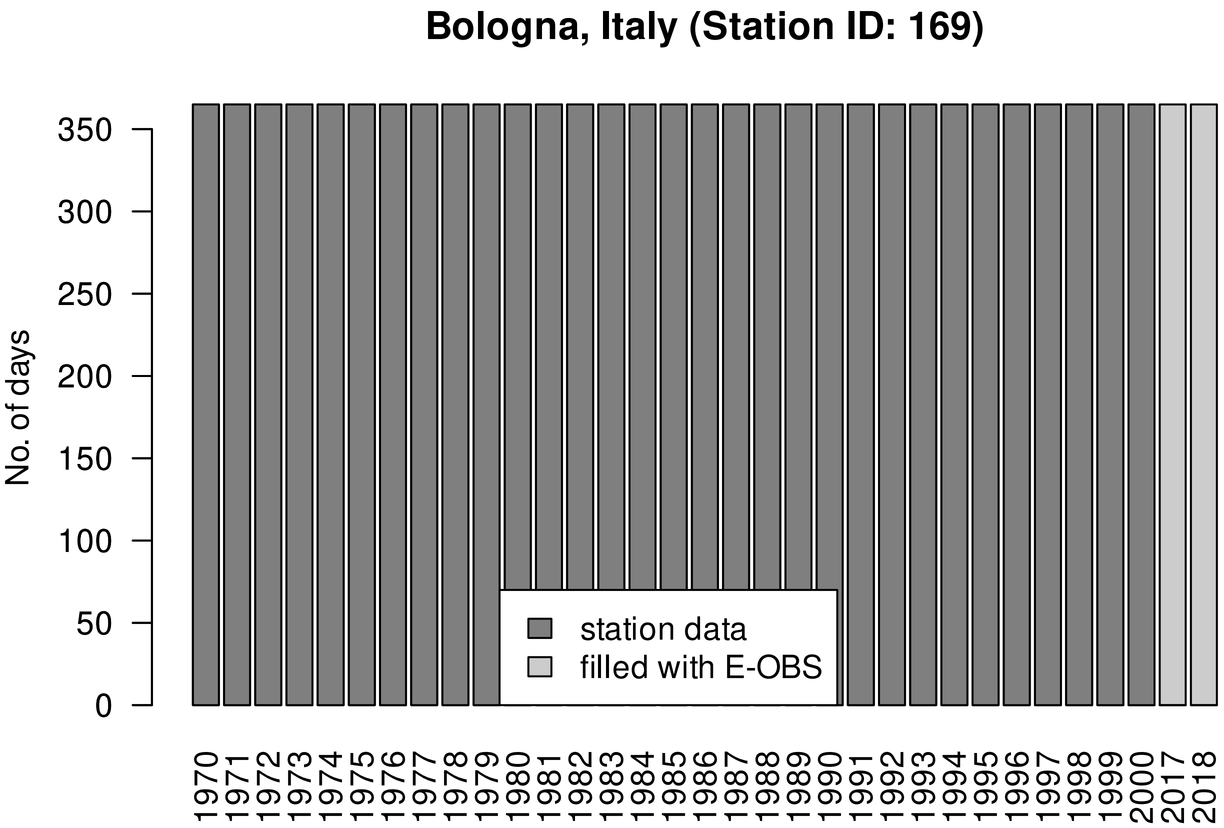

Bologna, Italy (Station ID: 169)
No. of days
0
50
100
150
200
250
300
350
station data
filled with E-OBS
1970
1971
1972
1973
1974
1975
1976
1977
1978
1979
1980
1981
1982
1983
1984
1985
1986
1987
1988
1989
1990
1991
1992
1993
1994
1995
1996
1997
1998
1999
2000
2017
2018

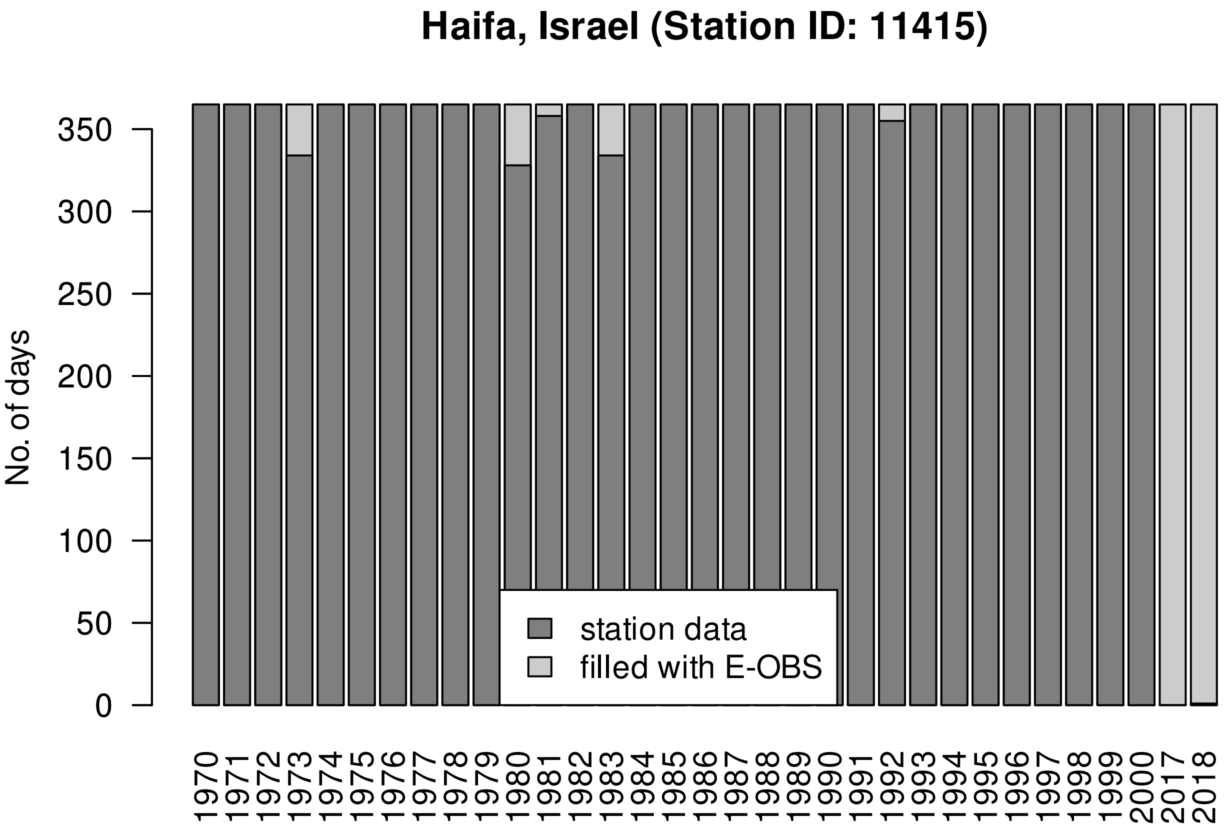

Haifa, Israel (Station ID: 11415)
No. of days
0
50
100
150
200
250
300
350
station data
filled with E-OBS
1970
1971
1972
1973
1974
1975
1976
1977
1978
1979
1980
1981
1982
1983
1984
1985
1986
1987
1988
1989
1990
1991
1992
1993
1994
1995
1996
1997
1998
1999
2000
2017
2018

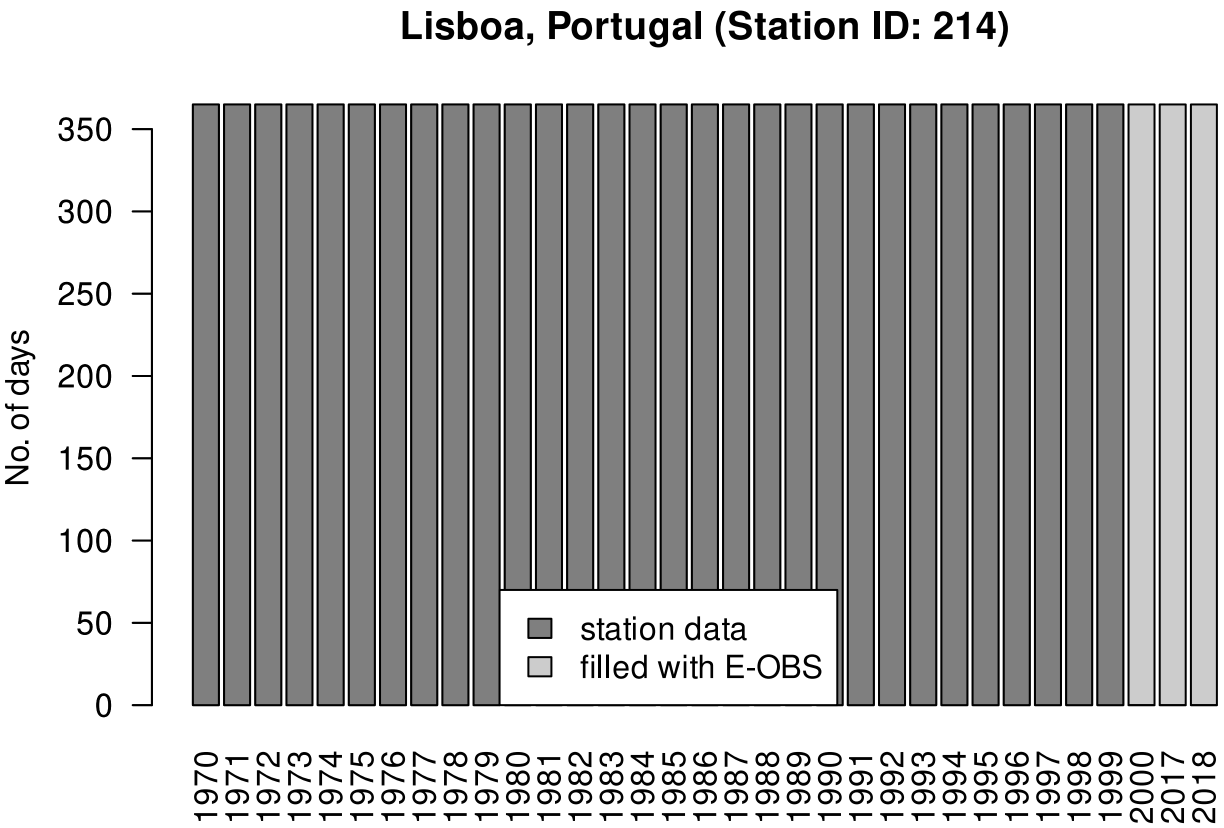
Lisboa, Portugal (Station ID: 214)
No. of days
0
50
100
150
200
250
300
350
station data
filled with E-OBS
1970
1971
1972
1973
1974
1975
1976
1977
1978
1979
1980
1981
1982
1983
1984
1985
1986
1987
1988
1989
1990
1991
1992
1993
1994
1995
1996
1997
1998
1999
2000
2017
2018

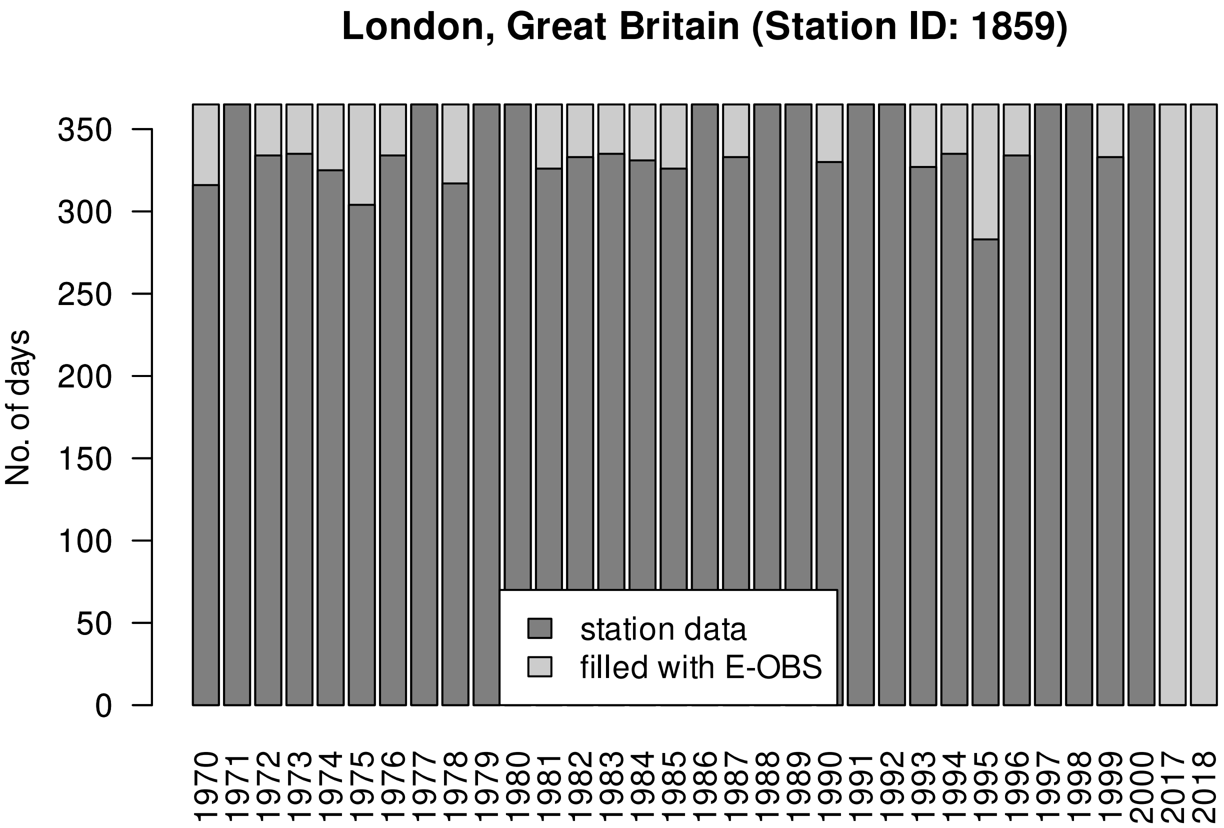
London, Great Britain (Station ID: 1859)
No. of days
0
50
100
150
200
250
300
350
station data
filled with E-OBS
1970
1971
1972
1973
1974
1975
1976
1977
1978
1979
1980
1981
1982
1983
1984
1985
1986
1987
1988
1989
1990
1991
1992
1993
1994
1995
1996
1997
1998
1999
2000
2017
2018

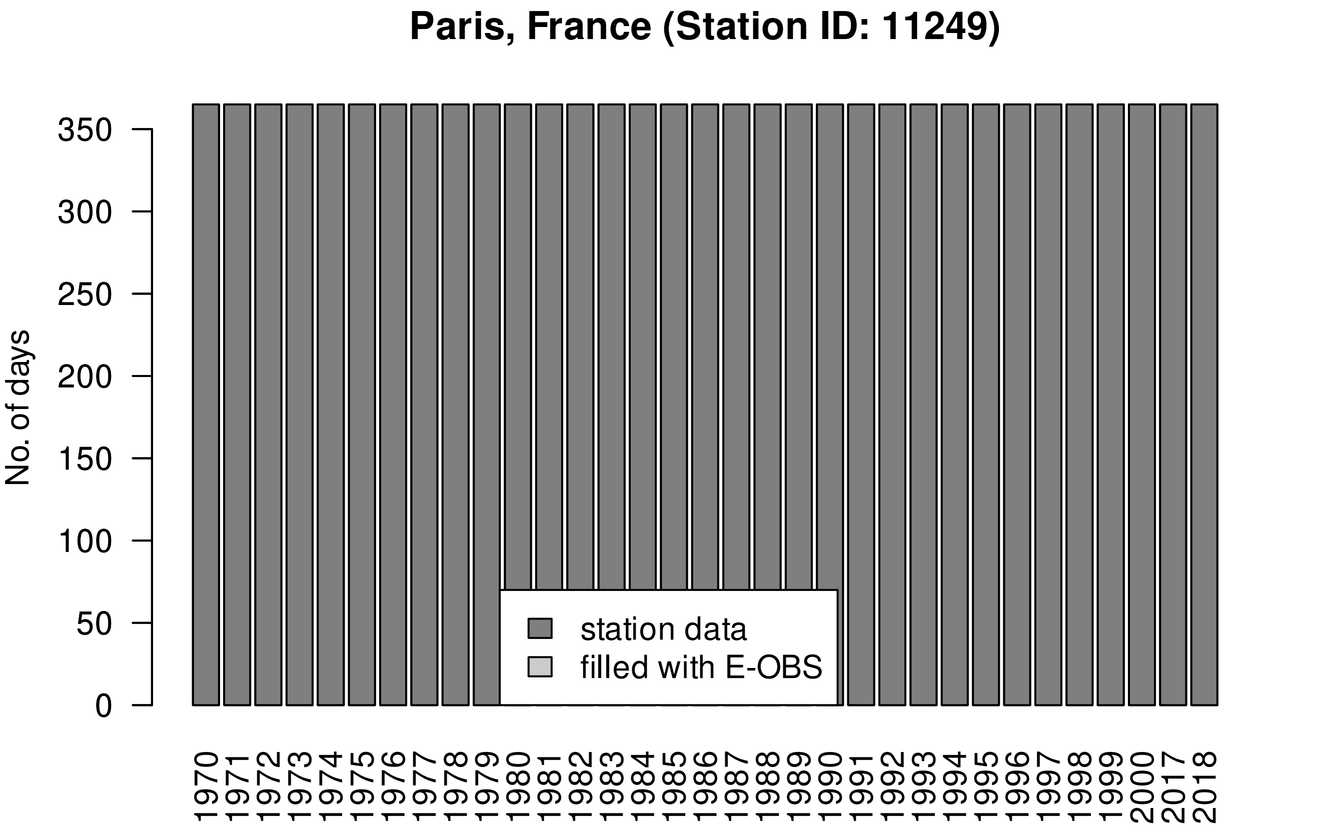

Paris, France (Station ID: 11249)
No. of days
0
50
100
150
200
250
300
350
station data
filled with E-OBS
1970
1971
1972
1973
1974
1975
1976
1977
1978
1979
1980
1981
1982
1983
1984
1985
1986
1987
1988
1989
1990
1991
1992
1993
1994
1995
1996
1997
1998
1999
2000
2017
2018

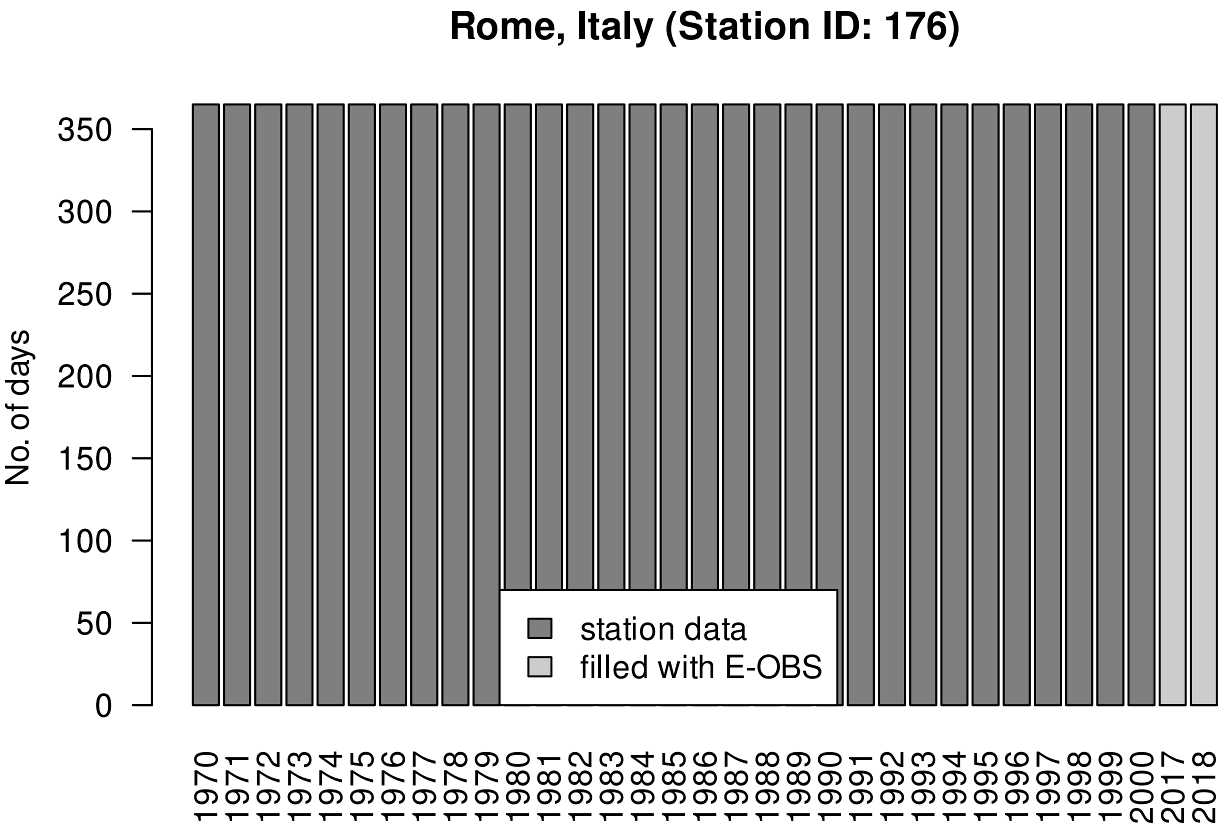

Rome, Italy (Station ID: 176)
No. of days
0
50
100
150
200
250
300
350
station data
filled with E-OBS
1970
1971
1972
1973
1974
1975
1976
1977
1978
1979
1980
1981
1982
1983
1984
1985
1986
1987
1988
1989
1990
1991
1992
1993
1994
1995
1996
1997
1998
1999
2000
2017
2018

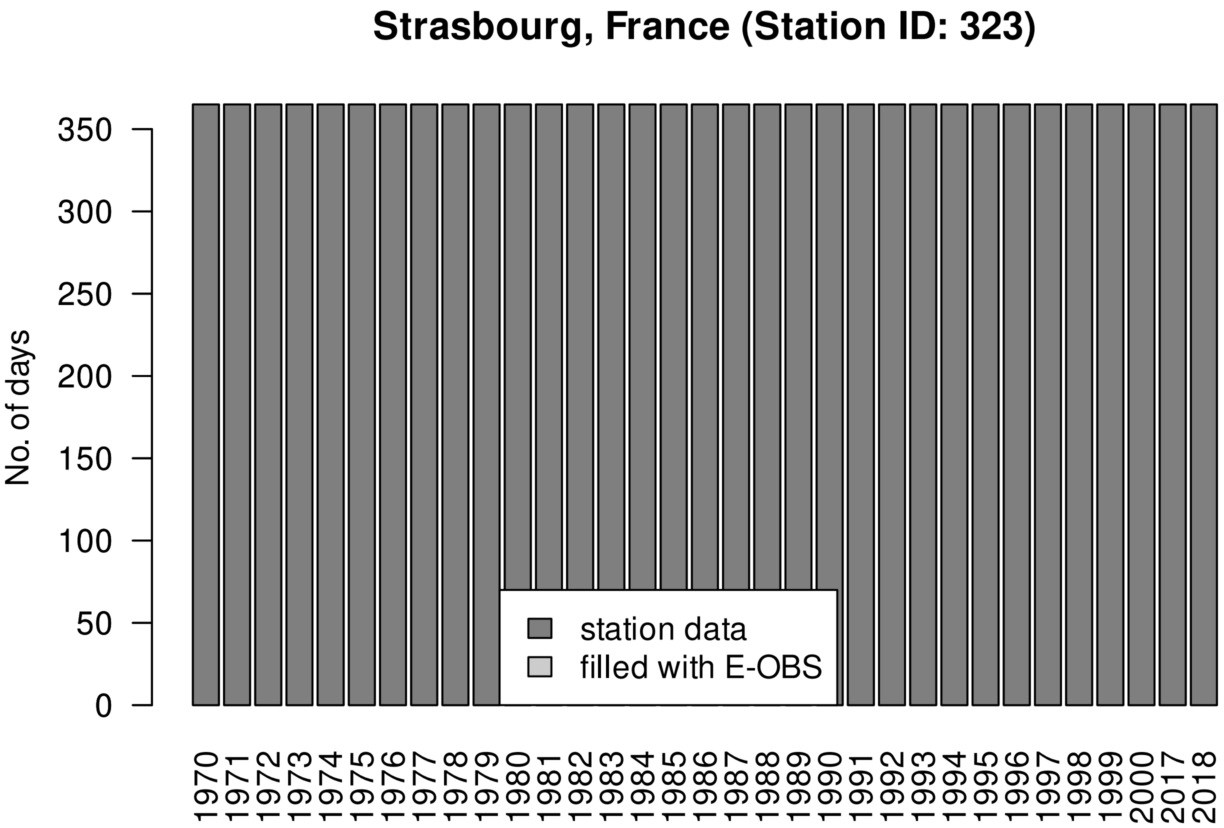
Strasbourg, France (Station ID: 323)
No. of days
0
50
100
150
200
250
300
350
station data
filled with E-OBS
1970
1971
1972
1973
1974
1975
1976
1977
1978
1979
1980
1981
1982
1983
1984
1985
1986
1987
1988
1989
1990
1991
1992
1993
1994
1995
1996
1997
1998
1999
2000
2017
2018

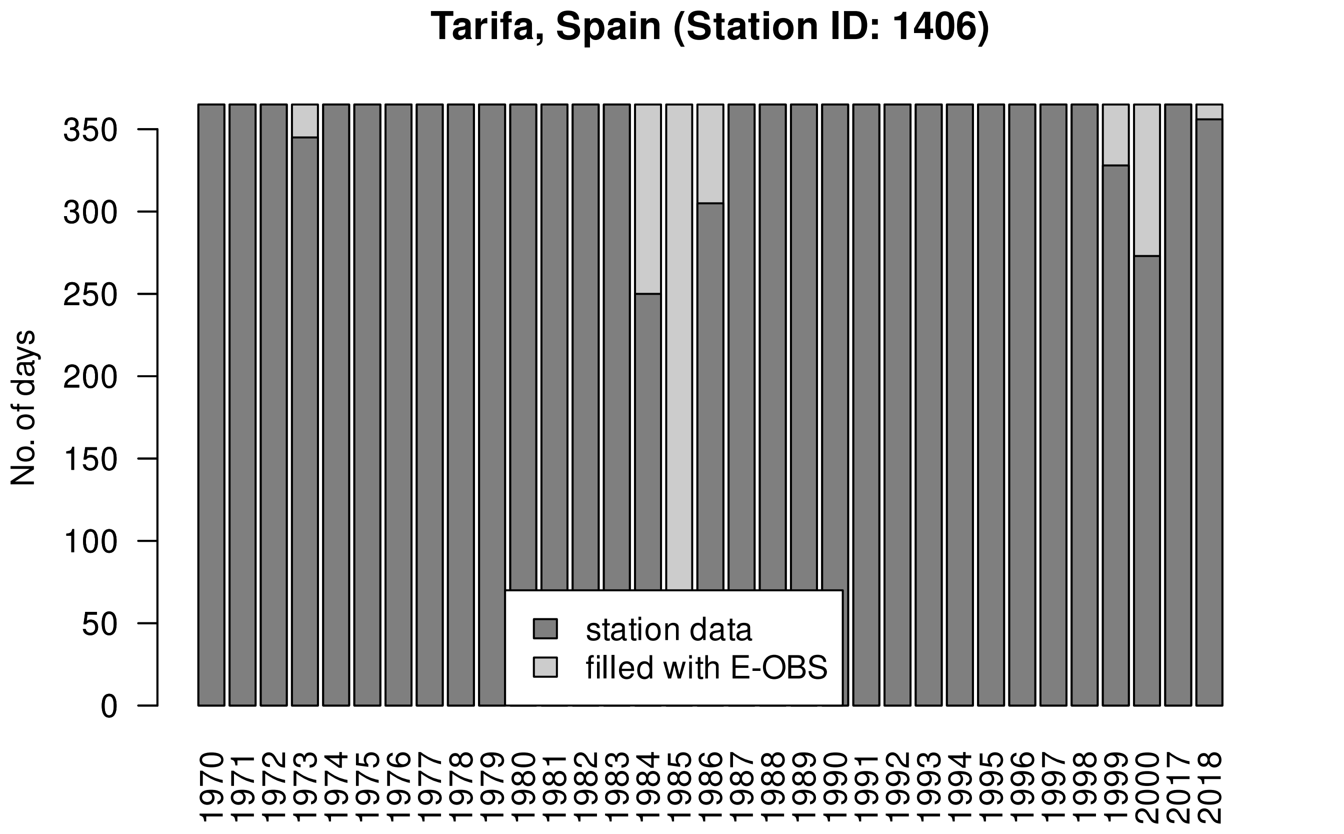
Tarifa, Spain (Station ID: 1406)
No. of days
0
50
100
150
200
250
300
350
station data
filled with E-OBS
1970
1971
1972
1973
1974
1975
1976
1977
1978
1979
1980
1981
1982
1983
1984
1985
1986
1987
1988
1989
1990
1991
1992
1993
1994
1995
1996
1997
1998
1999
2000
2017
2018

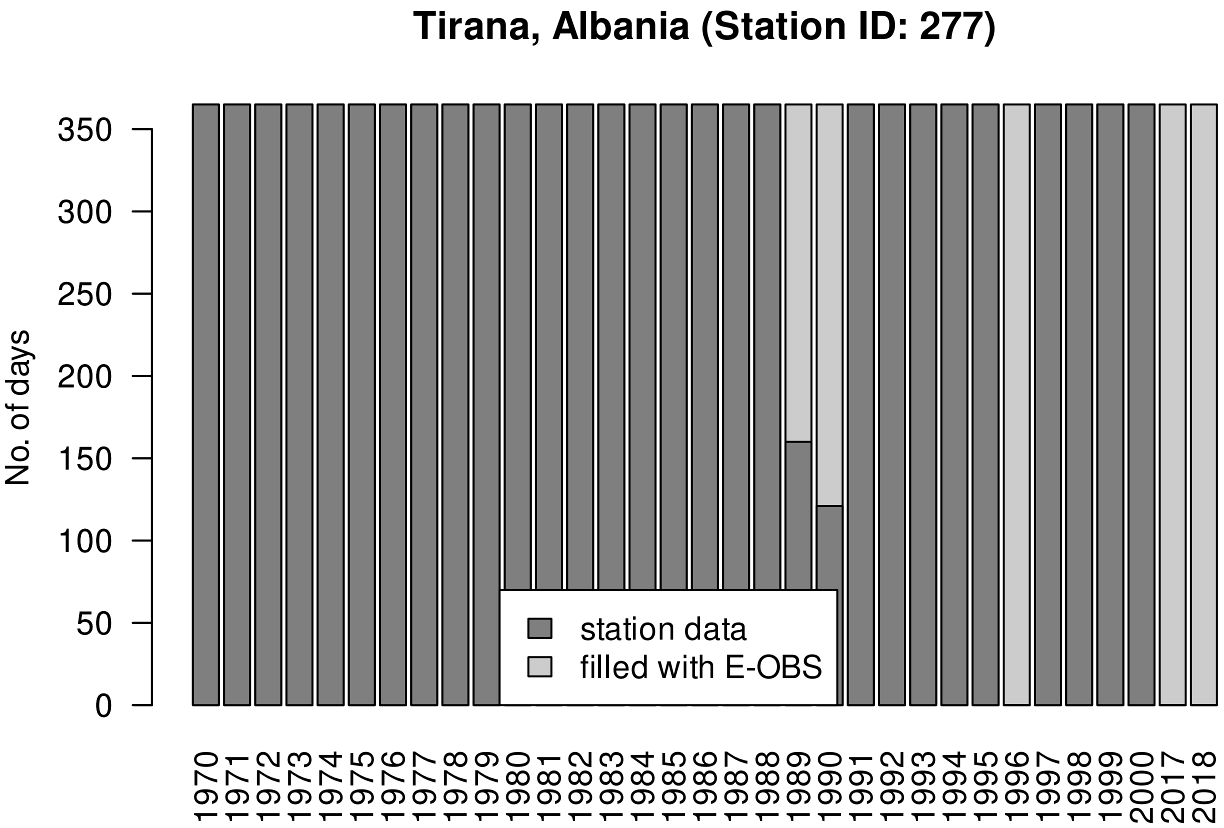

Tirana, Albania (Station ID: 277)
No. of days
0
50
100
150
200
250
300
350
station data
filled with E-OBS
1970
1971
1972
1973
1974
1975
1976
1977
1978
1979
1980
1981
1982
1983
1984
1985
1986
1987
1988
1989
1990
1991
1992
1993
1994
1995
1996
1997
1998
1999
2000
2017
2018

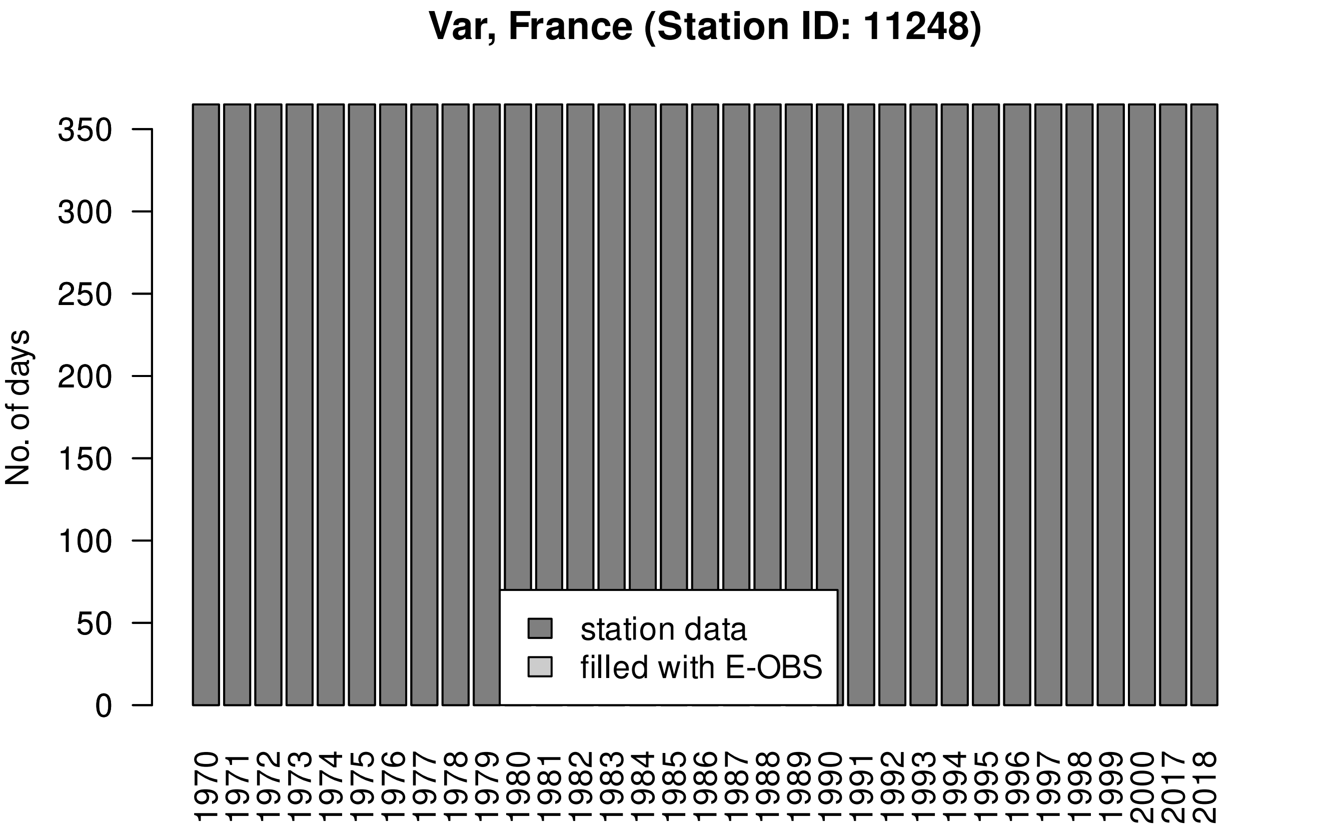
Var, France (Station ID: 11248)
No. of days
0
50
100
150
200
250
300
350
station data
filled with E-OBS
1970
1971
1972
1973
1974
1975
1976
1977
1978
1979
1980
1981
1982
1983
1984
1985
1986
1987
1988
1989
1990
1991
1992
1993
1994
1995
1996
1997
1998
1999
2000
2017
2018
