# Supplementary material for: Chikungunya Beyond the Tropics: Where and When Do We Expect Disease Transmission in Europe?
Source: Viruses. 2021 May 29;13(6):1024. doi: 10.3390/v13061024 (PMC8226708; doi:10.3390/v13061024)
Supplement: Supplementary file 1 [file viruses-13-01024-s001.zip › Figure S5.pdf]

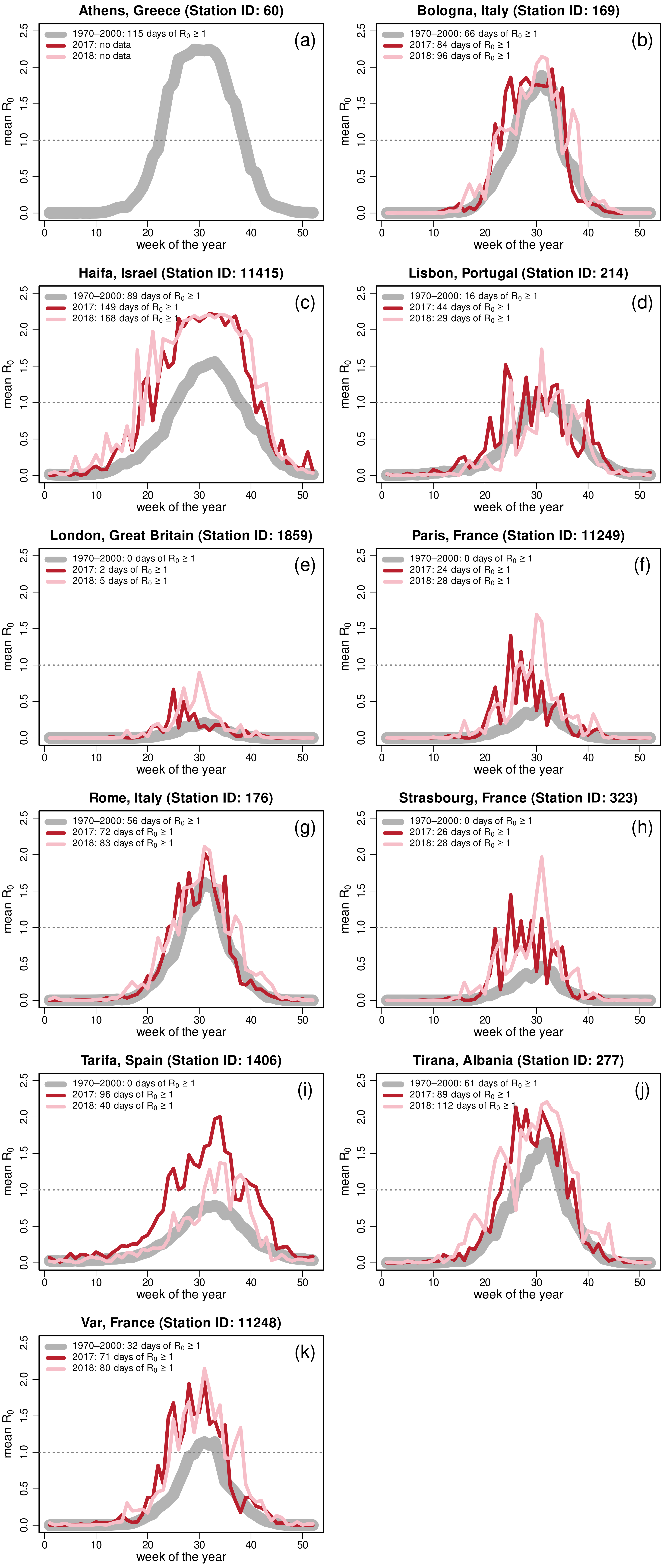
Athens, Greece (Station ID: 60)
(a)
1970–2000: 115 days of $R_0 \geq 1$
2017: no data
2018: no data
Bologna, Italy (Station ID: 169)
(b)
1970–2000: 66 days of $R_0 \geq 1$
2017: 84 days of $R_0 \geq 1$
2018: 96 days of $R_0 \geq 1$
Haifa, Israel (Station ID: 11415)
(c)
1970–2000: 89 days of $R_0 \geq 1$
2017: 149 days of $R_0 \geq 1$
2018: 168 days of $R_0 \geq 1$
Lisbon, Portugal (Station ID: 214)
(d)
1970–2000: 16 days of $R_0 \geq 1$
2017: 44 days of $R_0 \geq 1$
2018: 29 days of $R_0 \geq 1$
London, Great Britain (Station ID: 1859)
(e)
1970–2000: 0 days of $R_0 \geq 1$
2017: 2 days of $R_0 \geq 1$
2018: 5 days of $R_0 \geq 1$
Paris, France (Station ID: 11249)
(f)
1970–2000: 0 days of $R_0 \geq 1$
2017: 24 days of $R_0 \geq 1$
2018: 28 days of $R_0 \geq 1$
Rome, Italy (Station ID: 176)
(g)
1970–2000: 56 days of $R_0 \geq 1$
2017: 72 days of $R_0 \geq 1$
2018: 83 days of $R_0 \geq 1$
Strasbourg, France (Station ID: 323)
(h)
1970–2000: 0 days of $R_0 \geq 1$
2017: 26 days of $R_0 \geq 1$
2018: 28 days of $R_0 \geq 1$
Tarifa, Spain (Station ID: 1406)
(i)
1970–2000: 0 days of $R_0 \geq 1$
2017: 96 days of $R_0 \geq 1$
2018: 40 days of $R_0 \geq 1$
Tirana, Albania (Station ID: 277)
(j)
1970–2000: 61 days of $R_0 \geq 1$
2017: 89 days of $R_0 \geq 1$
2018: 112 days of $R_0 \geq 1$
Var, France (Station ID: 11248)
(k)
1970–2000: 32 days of $R_0 \geq 1$
2017: 71 days of $R_0 \geq 1$
2018: 80 days of $R_0 \geq 1$
mean R_0
week of the year
0.0 0.5 1.0 1.5 2.0 2.5
0 10 20 30 40 50
