# Supplementary material for: Chikungunya Beyond the Tropics: Where and When Do We Expect Disease Transmission in Europe?
Source: Viruses. 2021 May 29;13(6):1024. doi: 10.3390/v13061024 (PMC8226708; doi:10.3390/v13061024)
Supplement: Supplementary file 1 [file viruses-13-01024-s001.zip › Table S1.pdf]

Table S1. Parameter selection for the Ecological Niche Model.

Variable Description	ID	usage	reason(s) for dropping
Annual Mean Temperature	Bio 1	✔	
Mean Diurnal Range (Mean of monthly (max temp - min temp))	Bio 2	✗	derviative variable
Isothermality (BIO2/BIO7) (* 100)	Bio 3	✗	derviative variable
Temperature Seasonality (standard deviation *100)	Bio 4	✗	derviative variable
Max Temperature of Warmest Month	Bio 5	✗	jackknife
Min Temperature of Coldest Month	Bio 6	✔	
Temperature Annual Range (BIO5-BIO6)	Bio 7	✔	
Mean Temperature of Wettest Quarter	Bio 8	✗	referring to wettest/driest month or quarter
Mean Temperature of Driest Quarter	Bio 9	✗	referring to wettest/driest month or quarter
Mean Temperature of Warmest Quarter	Bio 10	✔	
Mean Temperature of Coldest Quarter	Bio 11	✔	
Annual Precipitation	Bio 12	✔	
Precipitation of Wettest Month	Bio 13	✗	referring to wettest/driest month or quarter
Precipitation of Driest Month	Bio 14	✗	referring to wettest/driest month or quarter, jackknife
Precipitation Seasonality (Coefficient of Variation)	Bio 15	✗	derviative variable, jackknife
Precipitation of Wettest Quarter	Bio 16	✗	referring to wettest/driest month or quarter
Precipitation of Driest Quarter	Bio 17	✗	referring to wettest/driest month or quarter, jackknife
Precipitation of Warmest Quarter	Bio 18	✔	
Precipitation of Coldest Quarter	Bio 19	✗	jackknife
