# Supplementary material for: Chikungunya Beyond the Tropics: Where and When Do We Expect Disease Transmission in Europe?
Source: Viruses. 2021 May 29;13(6):1024. doi: 10.3390/v13061024 (PMC8226708; doi:10.3390/v13061024)
Supplement: Supplementary file 1 [file viruses-13-01024-s001.zip › Table S2.pdf]

Table S2. Parameters used in the epidemiological model [1]. For detailed descriptions and references please refer to the original paper. T = daily mean temperature in °C.

Symbol	Parameter	Equation/Probability Distribution
α	daily biting rate	pert(0.19, 0.31, 0.39)
β_{HM}	probability of human-to-mosquito transmission	pert(0.37, 0.40, 0.95)
β_{MH}	probability of mosquito-to human transmission	pert(0.50, 0.65, 0.80)
V	duration of the human infectious period	gamma(κ = 30, θ = 0.2)
L	average mosquito lifespan	$L=1/\mu$
μ	average daily mosquito mortality	$\mu(T) = 1.33048 - 2.32772 \times 10^{-1} \times T + 1.68529 \times 10^{-2} \times T^2 - 5.61719 \times 10^{-4} \times T^3 + 7.91643 \times 10^{-6} \times T^4 - 2.72000 \times 10^{-8} \times T^5$
γ	Proportion of mosquitoes surviving the EIP	$\gamma = e^{-EIP/L}$
EIP	Extrinsic incubation period	$EIP = e^{(\log EIP_{28}) \times e^{\beta_T \times (T-28)}}$
EIP_{28}	CHIKV EIP at 28°C	gamma(κ = 9, θ = 0.667)
β_T	factor to convert known EIP characteristics of DENV for CHIKV	normal(-0.08, 0.02)
φ	Mosquito density per human	$\varphi = \varphi_{max} (L/L_{max})$
φ_{max}	Mosquito density per human under ideal weather conditions	gamma($\kappa = \left(\frac{2}{0.6}\right)^2, \theta = \left(\frac{2}{0.6^2}\right)^{-1}$)*
L_{max}	maximum mean lifespan of mosquitoes (days)	$L_{max} = 10.9$

* This is based on the "Description, assumptions, and references" column in Table 1 of [1] rather than the gamma(2, 0.4) from the "Sampling distribution" column, as the latter appears to be incorrect. That distribution does not have a mean of 2 and standard deviation of 0.4, and using it leads to unrealistically low values of R_0. We thus assumed that the textual description was correct and the mathematical formulation of the distribution contained a typing error.

Reference

1. Ng, V.; Fazil, A.; Gachon, P.; Deuymes, G.; Radojević, M.; Mascarenhas, M.; Garasia, S.; Johansson, M. A.; Ogden, N. H., Assessment of the probability of autochthonous transmission of chikungunya virus in Canada under recent and projected climate change. Environ. Health Perspect. 2017, 125, (6), 067001 doi.10.1289/Ehp669.
