# Supplementary figures and images for: Chikungunya Beyond the Tropics: Where and When Do We Expect Disease Transmission in Europe?
Source: Viruses. 2021 May 29;13(6):1024. doi: 10.3390/v13061024 (PMC8226708; doi:10.3390/v13061024)

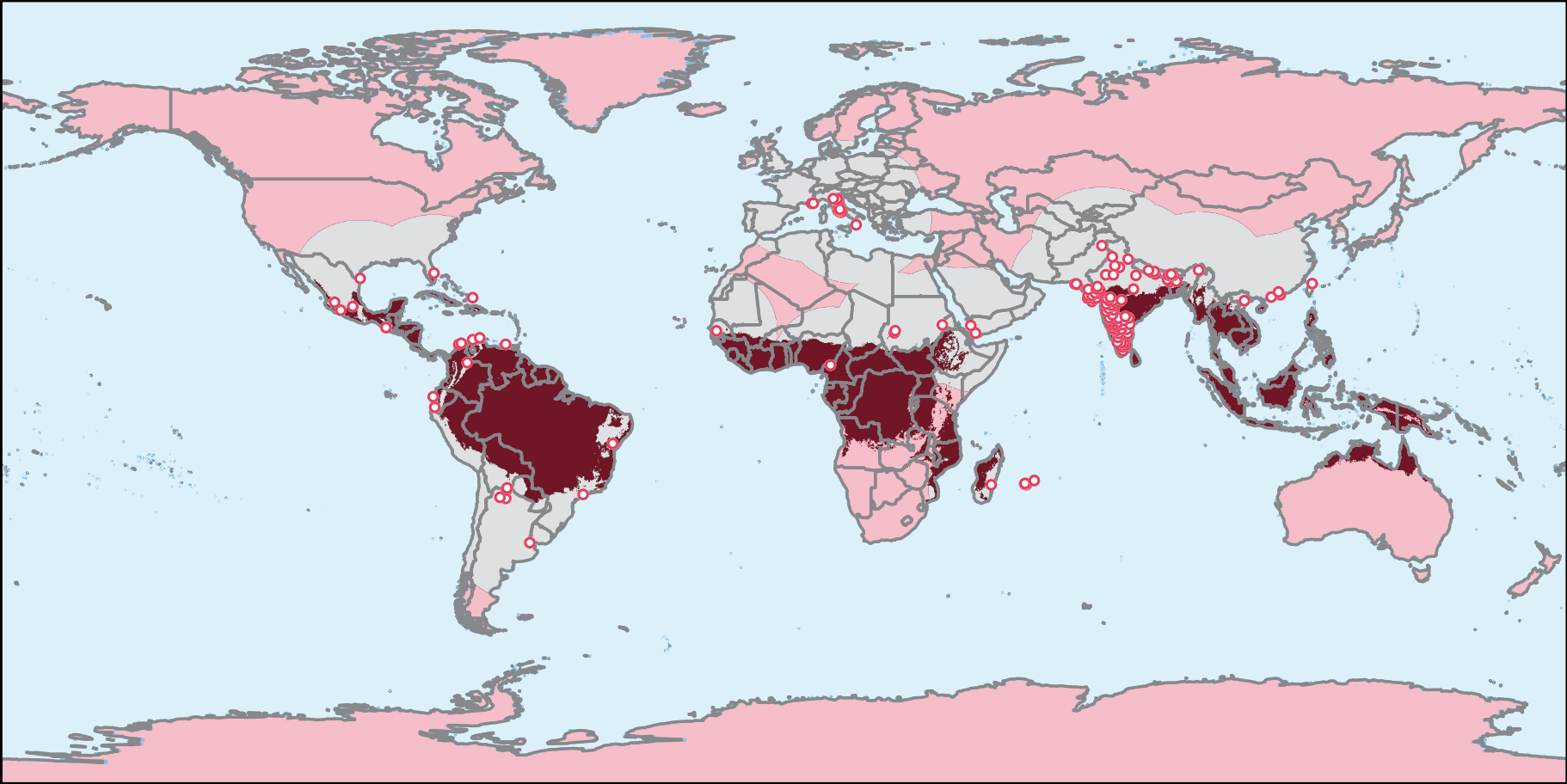

○ occurrence records final model    ■ excluded: tropical    ■ excluded: outside buffer    ■ calibration area

Supplement: Supplementary file 1 [file viruses-13-01024-s001.zip › Figure S1.pdf]

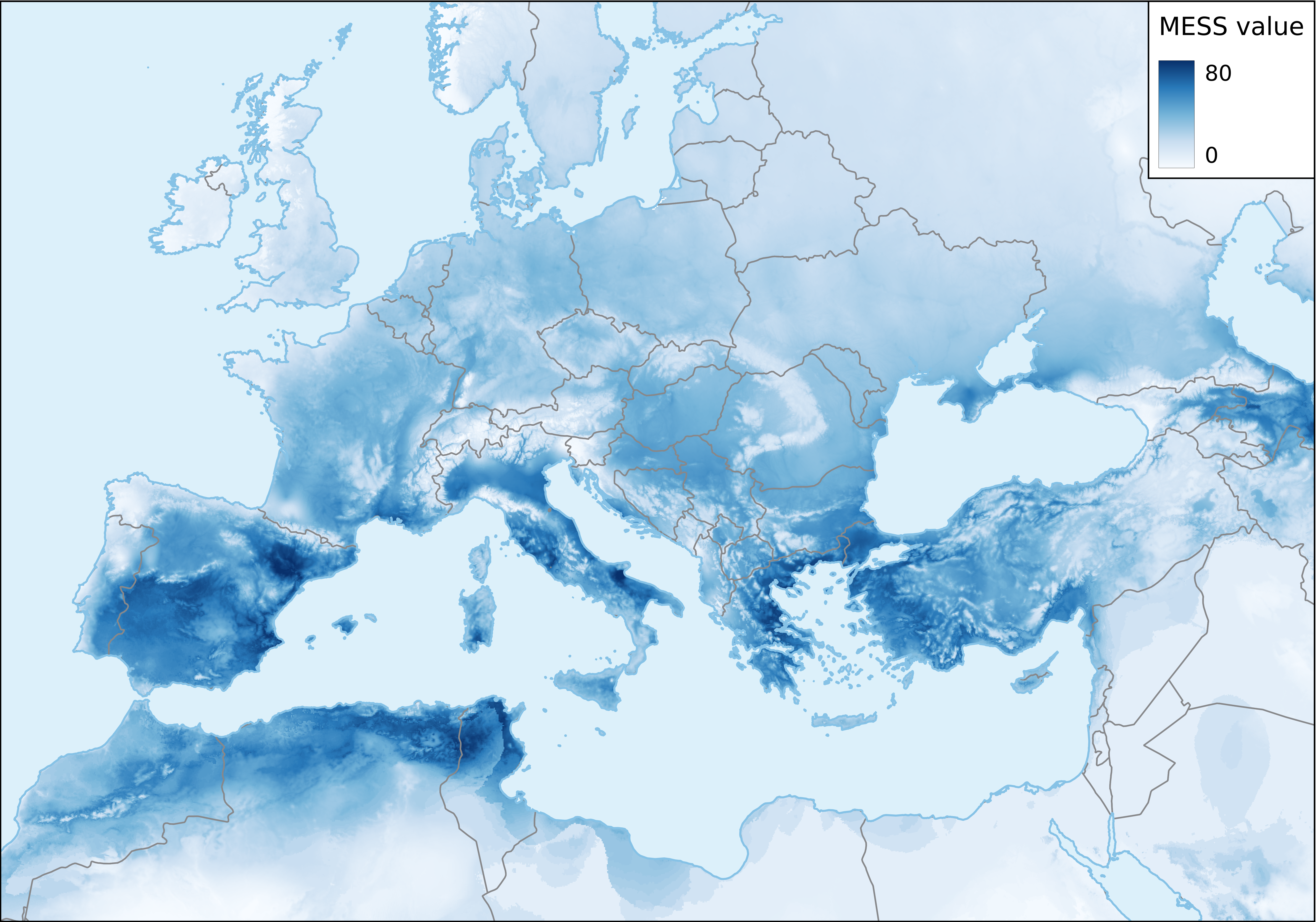

Supplement: Supplementary file 1 [file viruses-13-01024-s001.zip › Figure S2.png]
